# Bacteria and Fungi Synergistically Reprogram Flavonoid Metabolites in the Pericarp of *Citrus Reticulata* 'Chachi' During Storage

**DOI:** 10.1002/advs.202500267

**Published:** 2026-02-09

**Authors:** Jianmu Su, Sisheng Zhang, Lidi Liang, Haiyi Bai, Mei Bai, Hanjun He, Yayu Wang, Huan Liu, Xiangxiu Liang, Yu Sun, Hong Wu

**Affiliations:** ^1^ Centre For Medicinal Plant Research College of Life Sciences South China Agricultural University Guangzhou P. R. China; ^2^ Guangxi Health Science College Nanning P. R. China; ^3^ State Key Laboratory for Quality Ensurance and Sustainable Use of Dao‐di Herbs Beijing P. R. China; ^4^ Guangdong Key Laboratory for Innovative Development and Utilization of Forest Plant Germplasm South China Agricultural University Guangzhou P. R. China; ^5^ State Key Laboratory of Agricultural Genomics BGI‐Shenzhen Shenzhen P. R. China

**Keywords:** aging, citrus reticulata 'Chachi', microbiome, metabolites reprogram, polymethoxyflavones

## Abstract

The mature pericarp of *Citrus reticulata* ' Chachi ' (PCRC) is a traditional Chinese medicine known for its enhanced efficacy through long‐term storage and processing. However, the specific mechanisms underlying these enhancements remain unclear. This study employed widely targeted metabolomics, microbial amplicon sequencing, and fermentation assays to investigate the microbiome 's influence on PCRC 's flavonoid profile over 0–19 years of storage. Correlation analysis revealed that the accumulation of polymethoxyflavones (PMFs) was closely linked to specific bacterial and fungal communities. Solid‐state fermentation showed that *Bacillus subtilis* N18‐1 enhanced the content of certain PMFs, while *Aspergillus tubingensis* P21‐1 reduced them. Liquid‐state assays confirmed that *A. tubingensis* P21‐1 converted nobiletin to 3 ' ‐demethylnobiletin, and *B*. subtilis N18‐1 further converted this to tangeretin. Combined with genome sequencing and molecular docking, four candidate genes were identified. The catalytic activity verification assays demonstrated that At21‐68 and At21‐21 catalyze the conversion of nobiletin to 3 '‐demethylnobiletin, while Bs18‐51 and Bs18‐84 catalyze the conversion of 3 ' ‐demethylnobiletin to tangeretin. These findings highlight the synergistic molecular mechanism by which microorganisms modulate PMFs during PCRC aging, providing insights for optimizing medicinal plant aging through microbial biotransformation.

## Graphical abstract

The authors employed widely targeted metabolomics, microbial amplicon sequencing, and fermentation assays to investigate the microbiome 's influence on PCRC 's (pericarp of Citrus reticulata ' Chachi ') flavonoid profile over 0–19 years of storage. Based on the correlation analysis, solid‐state and liquid‐state fermentation, and catalytic activity verification assays, they reveal that At 21‐68, At 21‐21 catalyze the conversion of nobiletin to 3 '‐demethylnobiletin, while Bs18‐51 and Bs18‐84 catalyze the conversion of 3 '‐demethylnobiletin to tangeretin.

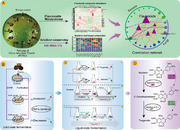

## 1 Introduction

The mature pericarp of *Citrus reticulata* ' Chachi ' (PCRC) is a traditional Chinese medicine known for its enhanced efficacy through long‐term storage and processing [1, 2]. Extensive phytochemical studies have revealed that PCRC primarily contains flavonoids [3], essential oils [4], polysaccharides [5], and alkaloids [6]. The flavonoids in PCRC consist mainly of flavonoid glycosides and Polymethoxylated flavonoids (PMFs) [7, 8, 9]. Modern pharmacological research has confirmed that flavonoids exhibit various beneficial effects, such as antioxidative, antibacterial, antiviral, anti‐inflammatory, antitumor, and hypolipidemic properties [10, 11]. Notably, PMFs have been shown to possess significant biological activities, including inhibition of chronic inflammation [12], cancer prevention [13, 14], antiatherogenic effects [15], and modulation of the gut microbiota, thereby protecting against metabolic syndrome [11]. Consequently, PMFs play a crucial role in determining the efficacy of PCRC and have been designated as key components for its quality evaluation in the Pharmacopoeia of the People's Republic of China [1].

For hundreds of years, PCRC has been considered to have better efficacy and fewer side effects after aging by storage in shaded and dry place [5, 16]. In recent years, several studies have demonstrated significant changes in flavonoids, total phenols, carotenoids, and antioxidant activity during the aging process of PCRC [16, 17]. The content of hesperidin decreased while the levels of 5 PMFs increased in PCRC during a 36‐month storage period at 25°C [17]. Luo et al. identified 31 metabolites that distinguished PCRC based on different storage years [18]. Liang et al. found that the aging group had higher levels of 15 PMFs during PCRC storage [19].

In recent years, researchers have found that microbial communities are key factors influencing the quality maturation of PCRC during storage. Traditional culture‐dependent methods revealed that Bacillaceae are the dominant culturable bacteria in PCRC. For example, Yang et al. isolated a total of 23 bacterial strains from 7‐year‐old PCRC samples, all belonging to the *Bacillus* and *Paenibacillus* genera [20]. Most microorganisms are difficult to culture, and microbial communities display countless spatiotemporal dynamic patterns, collectively known as “microbial dark matter” [21]. High‐throughput sequencing further identified dominant bacterial genera, including Lactococcus in PCRC, with some bacteria directly associated with bioactive components. *Bacillus* and *Lactococcus* were confirmed to be closely related to the accumulation of flavonoids such as nobiletin [22, 23]. Previous studies reported that bacterial genera *Herbaspirillum*, *Sphingomonas*, *Streptococcus*, *Pseudomonas*, and *Escherichia Shigella* showed strong connections with volatile components, and fungal genera *Exobasidium*, *Xeromyces*, *Pseudocercospora*, *Russula*, and *Aspergillus*, showed strong connections with volatile components in Citri Reticulatae Pericarpium [24, 25].

Existing studies suggest a correlation between the accumulation of flavonoid and volatile components and the microbial communities in aged PCRC. However, correlation does not imply causation. These studies lack microbial isolation or the absence of metabolic pathway validation, which significantly hinders the understanding of the underlying biological processes of microorganisms in the influence of flavonoid content during aging. Therefore, there is a critical need to comprehensively elucidate the direct effects and pathways by which microorganisms modulate flavonoid profiles.

To address these limitations, we employed multi‐omics approaches, including bacterial 16S rRNA, fungal ITS amplicon sequencing, and widely targeted metabolomics, to identify the key microorganisms on PCRC metabolism during the 19 years of aging process. We further isolated and cultured 243 bacterial and fungal strains from PCRC and selected 10 strains to conduct solid‐state and liquid‐state fermentation assays to verify their role in flavonoid transformation. We found two key strains *Bacillus subtilis* N18‐1 and *Aspergillus tubingensis* P21‐1, synergistically reprogrammed the flavonoid transformation from nobiletin to tangeretin. By sequencing the genomes of the two key strains, we identified potential genes involved in PMFs transformation. The catalytic activity verification assays demonstrate that At21‐68 and At21‐21 catalyzed the conversion of nobiletin to 3'‐demethylnobiletin, while Bs18‐51 and Bs18‐84 catalyzed the conversion of 3'‐demethylnobiletin to tangeretin. Our findings elucidate the mechanisms by which microorganisms synergistically reprogrammed the PMFs transformation during aging, providing insights into leveraging microbial biotransformation to optimize and control the aging process of medicinal plants.

## 2 Results

### 2.1 Metabolism Features of Flavonoids During Storage

**FIGURE 1:**
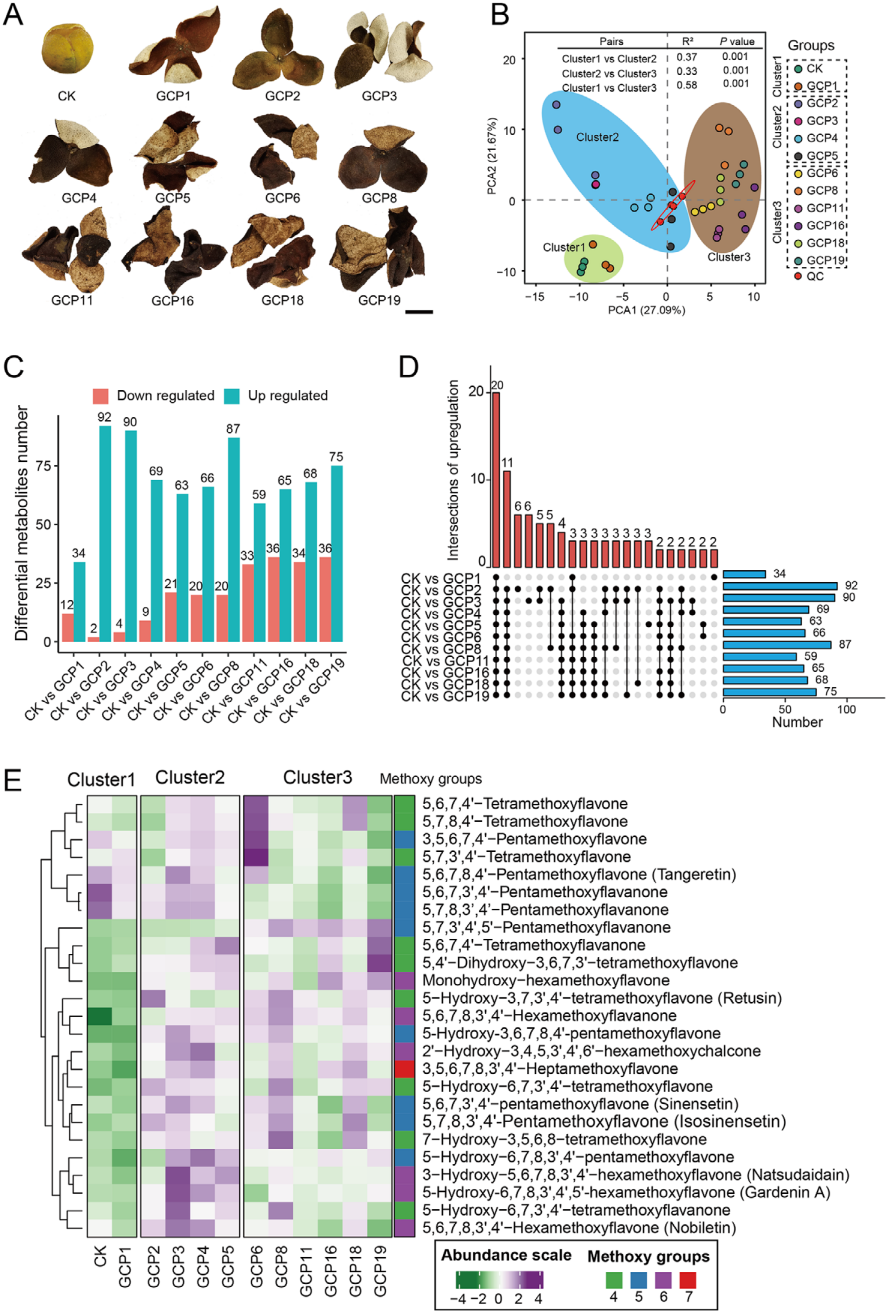
Metabolite features of flavonoids in 12 PCRC groups. (A) Photographs showing the appearance of PCRC in different storage years. Scale bar = 2 cm. (B) Principal Component Analysis (PCA) of flavonoid metabolites in 12 PCRC groups, which including freshly harvested pericarps (CK), 1‐year‐old pericarps (GCP1), 2‐year‐old pericarps (GCP2), 3‐year‐old pericarps (GCP3), 4‐year‐old pericarps (GCP4), 5‐year‐old pericarps (GCP5), 6‐year‐old pericarps (GCP6), 8‐year‐old pericarps (GCP8), 11‐year‐old pericarps (GCP11), 16‐year‐old pericarps (GCP16), 18‐year‐old pericarps (GCP18), 19‐year‐old pericarps (GCP19). Three biological replicates were included in each storage year. PC1 is represented on the horizontal axis, and PC2 on the vertical axis. Samples are color‐coded according to their group information. The significance of clustering was tested using PERMANOVA, the Adonis function in R, which yielded the R^2^ value and the corresponding *p* value. QC represents QC samples, which were combined 20 µL from each sample to create a mixed sample. During the testing process, we analyzed the mixed sample after every 9 individual samples, which generated 4 QC samples. (C) The number of downregulated and upregulated metabolites in eleven pairwise group comparisons. (D) Up‐set diagram showing the intersections of upregulated metabolites among the eleven pairwise group comparisons. The upper bar plot represents the number of intersecting upregulated metabolites among groups. The bar plot on the right represents the numbers of intersecting upregulated metabolites in each group. (E) Abundance of PMFs in all groups. Samples were clustered into three clusters based on principal component analysis. The total metabolite ion intensity of the 25 PMFs was scaled and centered for comparison. In the heatmap, colors represent relative abundance: green (low), white (moderate), and purple (high). Methoxy groups represent the number of methoxy in each PMF.

To investigate the impact of aging on the flavonoid profile of PCRC, a widely targeted metabolomics analysis was conducted on 36 PCRC samples across 12 groups, including freshly harvested pericarps (CK), 1‐year‐old pericarps (GCP1), 2‐year‐old pericarps (GCP2), 3‐year‐old pericarps (GCP3), 4‐year‐old pericarps (GCP4), 5‐year‐old pericarps (GCP5), 6‐year‐old pericarps (GCP6), 8‐year‐old pericarps (GCP8), 11‐year‐old pericarps (GCP11), 16‐year‐old pericarps (GCP16), 18‐year‐old pericarps (GCP18), 19‐year‐old pericarps (GCP19) (Figure 1A). A total of 212 flavonoid metabolites were detected, including 100 flavonoids, 46 flavonols, 26 flavonoid C‐glycosides, 18 dihydroflavones, 10 tannins, 3 flavanols, and 5 dihydroflavonols (Table S1). Clustering analysis revealed that samples within the same age group tended to cluster together, while samples from different groups were clearly separated (Figure S1). Principal component analysis (PCA) further confirmed the distinct clustering patterns, categorizing all samples into three clusters: CK and GCP1 formed one cluster; GCP2, GCP3, GCP4, and GCP5 formed another cluster; and GCP6, GCP8, GCP11, GCP16, GCP18, and GCP19 formed the third cluster (Figure 1B). Similarity test results indicated significant differences among the three clusters, with the difference between cluster 1 and cluster 3 (R^2^ = 0.58, *p* value = 0.001) being larger than that between cluster 2 and cluster 3 (R^2^ = 0.33, *p* value = 0.001) (Figure 1B). Some variation among samples from the same year was also observed. For instance, two samples from GCP3 are closer to GCP5, while one sample from GCP3 is closer to GCP4 (Figure 1B).

By comparing the ion intensity of flavonoids in PCRC samples at different aging periods, we identified a total of 46 differential metabolites in the comparisons between CK and GCP1, with 34 showing increased levels and 12 showing decreased levels in GCP1. Additionally, 94 metabolites were found to have increased (92) or decreased levels (2) in the comparison of CK and GCP2 (Figure 1C and Figure S2). A similar pattern was observed in the remaining comparisons, with the number of up‐regulated flavonoids in aging groups always higher than in CK. Notably, 20 metabolites were found to be co‐upregulated in all 11 groups of PCRC samples (Figure 1D and Figure S3). Among these metabolites, two were PMFs (5,7,8,3',4'‐Pentamethoxyflavanone and 5‐hydroxy‐3,7,4'‐trimethoxyflavone), six were PMFs precursors (apigenin, 5,7,4'‐trihydroxyflavanone, luteolin, eriodictyol, 4',5‐dihydroxy‐3',5'‐dimethoxyflavone, and 5,7,4'‐trihydroxy‐8,3'‐dimethoxyflavonol), and the remaining 12 were other flavonoids. Interestingly, hesperetin‐7‐O‐glucoside, hesperetin‐5,7‐di‐O‐glucoside, and gallocatechin were exclusively identified in aged PCRC samples (Figure S4). This finding suggests that these three flavonoids may be formed during the aging process of PCRC and can serve as marker components to differentiate fresh PCRC from aged PCRC.

In addition, 46 flavonoids carrying two or more methoxy groups were identified. Among these, 25 PMFs carrying four or more methoxy groups were found in all samples. The total metabolite ion intensity of the 25 PMFs showed an overall increase in PMFs content during storage (Figure 1E). Specifically, 21 out of the 25 PMFs increased in content, while only 4 PMFs decreased during the storage period. The abundance of 5‐Hydroxy‐6,7,8,3',4'‐pentamethoxyflavone was found to increase from 3 years of storage. Some PMFs like nobiletin, tangeretin, natsudaidain, gardenin A, 5‐Hydroxy‐6,7,3',4'‐tetramethoxyflavanone reached their highest abundance at 3 years of aging. Other PMFs like 5,6,7,4'‐Tetramethoxyflavone, 5,7,8,4'‐Tetramethoxyflavone, 3,5,6,7,4'‐Pentamethoxyflavone, and 5,7,3',4'‐Tetramethoxyflavone reached their highest abundance at 6 years of aging. In contrast, some PMFs only reached their highest value after aging for up to 19 years, such as 5,6,7,4'‐Tetramethoxyflavanone, 5,4'‐Dihydroxy‐3,6,7,3'‐Tetramethoxyflavone, and monohydroxy‐Hexamethoxyflavone. These results imply that the content of most PMFs increases after aging for three or more years.

### 2.2 Microbial Taxonomy and Functional Composition in PCRC

To investigate the impact of the PCRC microbiome on flavonoid content, we first analyzed the bacterial composition of PCRC over different storage years through 16S rRNA gene sequencing. After dereplication and removal of chimeras, sequences were clustered into OTUs at 97% identity, resulting in a total of 1,229 OTUs across all samples. Nonbacterial, mitochondrial, and low‐abundance OTUs were eliminated, resulting in 419 final OTUs. The bacterial species accumulation curves demonstrated that our sample size had reached saturation, providing sufficient bacterial information about PCRC (Figure S5).

**FIGURE 2:**
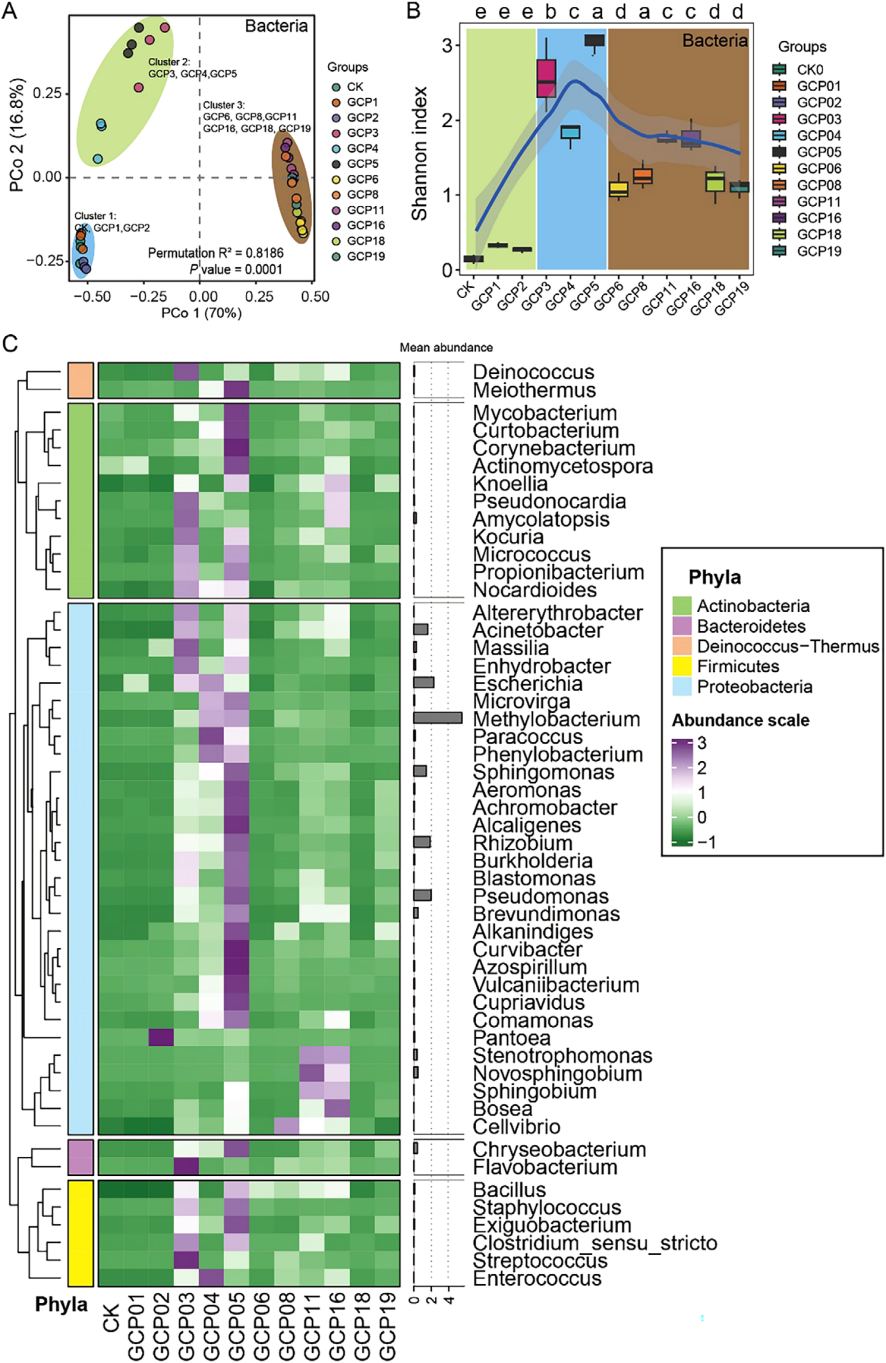
Core bacterial compositions in pericarp of *C. Reticulata* 'Chachi'. (A) Unconstrained principal coordinate analysis (PCoA) based on the bacterial composition. The *p*‐value and R2 were calculated by comparing the observed phylogenetic distance based on 999 permutations of a null model. (B) Shannon index of bacterial composition in different storage years. Different lowercase letters denote significance at *p* value < 0.05 (LSD Multiple Test). The background color was based on the cluster result of PCoA. The fitting curve is the mean fitting of the Shannon index. (C) Abundance of core bacterial genera. Abundances were scaled and centered. The bar plot on the right represents the mean abundance of each genus in all samples.

We assessed the influence of aging on the PCRC bacterial structure using UniFrac distance. Clustering analysis revealed that the 12 groups representing various aging periods formed three distinct classes. CK, GCP1, and GCP2 clustered together, while GCP3, GCP4, and GCP5 formed another cluster. The remaining groups (GCP6, GCP8, GCP11, GCP16, GCP18, and GCP19) constituted the third cluster (Figure 2A). Permutational multivariate analysis of variance (PERMANOVA) corroborated that the entire bacterial compositions were significantly different among the three clusters (*p* value < 0.001) (Figure 2A). The Shannon index, which measures bacterial composition diversity, was lowest in CK. GCP1 and GCP2 showed no significant differences compared to CK, while GCP3 to GCP19 exhibited significantly higher values than CK (Figure 2B). Notably, GCP5 had the highest Shannon index among all aging groups, followed by GCP3 and GCP4. Overall, the bacterial Shannon index demonstrated an initial increase followed by stabilization, indicating an overall increase in bacterial community diversity during PCRC storage from 3 to 19 years.

A total of 13 bacterial phyla and 163 bacterial genera were identified. Among these, Proteobacteria were the most abundant bacterial phyla recovered from all samples, ranging from 70.79% to 98.04% of the total sequence abundance, followed by Firmicutes (0%–18.06%) and Actinobacteria (0.32%–9.65%) (Figure S6). We identified 51 core bacterial genera that were present in more than 50% of the samples in each group (Figure 2C). The abundance of these core bacterial genera was significantly lower in CK, GCP1, and GCP2 compared with GCP3 to GCP19. Specifically, during the 3 to 5 years of storage, the abundance of core bacterial genera, including *Methylobacterium*, *Pseudomonas*, and *Sphingomonas*, reached their highest relative abundance and then exhibited a sharp decrease from GCP6 to GCP19 (Figure 2C and Figure S7). Overall, these findings suggest that these core bacterial genera are present at low abundance in the CK and GCP1‐2 groups but demonstrate a rapid increase from GCP3, reaching a peak in GCP5, followed by a sharp decline and eventual stabilization at a relatively constant level.

The functional composition of bacteria in PCRC was predicted. A total of 8,159 KOs (KEGG Orthology) were predicted, among which 23 O‐methyltransferases (OMTs) involved in flavonoid synthesis were identified. A Spearman correlation was calculated between the abundance of PMFs and OMTs to investigate the relationship between bacterial functional genes and PMFs biosynthesis (Figure S8). Eight OMTs showed positive correlation with 6 PMFs, including 5‐Hydroxy‐6,7,8,3',4'‐pentamethoxyflavone, 5,7,8,3',4'‐Pentamethoxyflavanone. This correlation suggests that changes in bacterial functional genes in PCRC are correlated with changes in PMFs.

**FIGURE 3:**
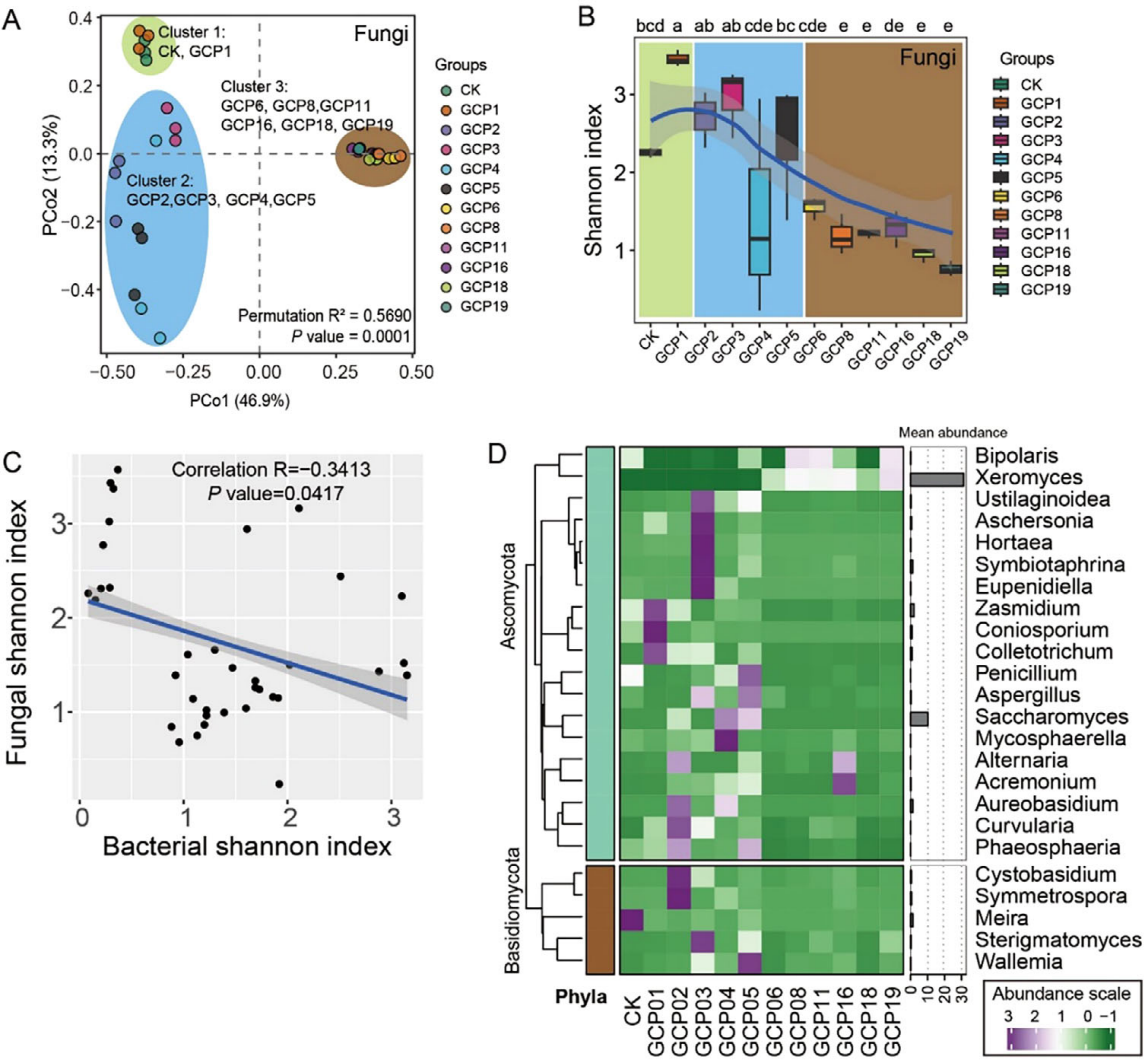
Core fungal compositions in pericarp of *C. Reticulata* 'Chachi'. (A) Unconstrained principal coordinate analysis (PCoA) based on fungal composition. (B) Shannon index of bacterial composition in different storage years. Different lowercase letters denote significance at *p*‐value < 0.05 (LSD Multiple Test). The background color was based on the cluster result of PCoA. The fitting curve is the mean fitting of the Shannon index. (C) Spearman correlation between bacterial and fungal Shannon indices. “Correlation R” represents Spearman's rank correlation coefficient. (D) Mean abundance of core fungal genera in each group, each including three biological replicates. Mean Abundance was scaled and centered. The bar plot on the right represents the mean abundance of fungi in all samples.

For fungal species, accumulation curves also indicated that our sample size was sufficient to capture most of the fungal information in the PCRC (Figure S9). Clustering the samples from different aging groups revealed that the 12 groups were divided into three distinct classes. CK and GCP1 formed one class, GCP2, GCP3, GCP4, and GCP5 clustered together in another class, and GCP6, GCP8, GCP11, GCP16, GCP18, and GCP19 formed the third class (Figure 3A). Interestingly, the Shannon index, a measure of fungal diversity, showed an increase from CK (fresh) to 1‐year storage period, followed by a decrease from 2 years to 19 years of storage (Figure 3B). Additionally, bacterial and fungal Shannon indices were negatively correlated (Figure 3C), indicating an inverse relationship between fungal and bacterial diversity within the PCRC during storage.

We identified a total of 3 fungal phyla and 54 fungal genera. The mean relative abundance of Ascomycota was the highest (87.5%–93.6%) and gradually increased during storage. In contrast, the abundance of Basidiomycota (0.6%–49%) gradually decreased with increasing storage age (Figure S10). We defined 24 core fungal genera that were present in more than 50% of the samples in each group (Figure 3D). Among the top 20 abundant fungal genera, *Xeromyces* exhibited the highest abundance, with a sharp increase at GCP6 and maintained the high abundance until the 19th year of storage (Figure 3D and Figure S11). *Saccharomyces* sharply increased from GCP2 to GCP5, except for GPC4. *Zasmidium* had a relatively high abundance from CK to GCP2, then experienced a continuous reduction in GPC3‐5 and GPC6‐19, respectively (Figure S11). *Meria* exhibited the highest abundance in fresh peel but remained at a low level in other storage samples.

Overall, bacterial diversity generally showed an upward trend, while fungal diversity showed a downward trend throughout the storage period. The abundance of bacterial genera *Methylobacterium*, *Pseudomonas*, and *Sphingomonas* peaked between 3 and 5 years of storage (Figure S7). The abundance of fungal genera *Saccharomyces*, *Zasmidium*, and *Meria* decreased during the storage period. These findings suggest that bacteria and fungi potentially have distinct contributions to the compositional changes in PCRC.

### 2.3 Correlation Between Microorganisms and Metabolites in PCRC

To investigate the relationship between microorganisms and metabolite components, we established a correlation‐based network. Filtering the correlation with a threshold (R value > 0.5 and *p* value < 0.05) resulted in 88 metabolites, 49 bacterial genera, and 20 fungal genera included in the network (Table S2). Among these, 46 bacterial genera exhibited positive correlations with metabolites, while 17 fungal genera demonstrated negative correlations.

**FIGURE 4:**
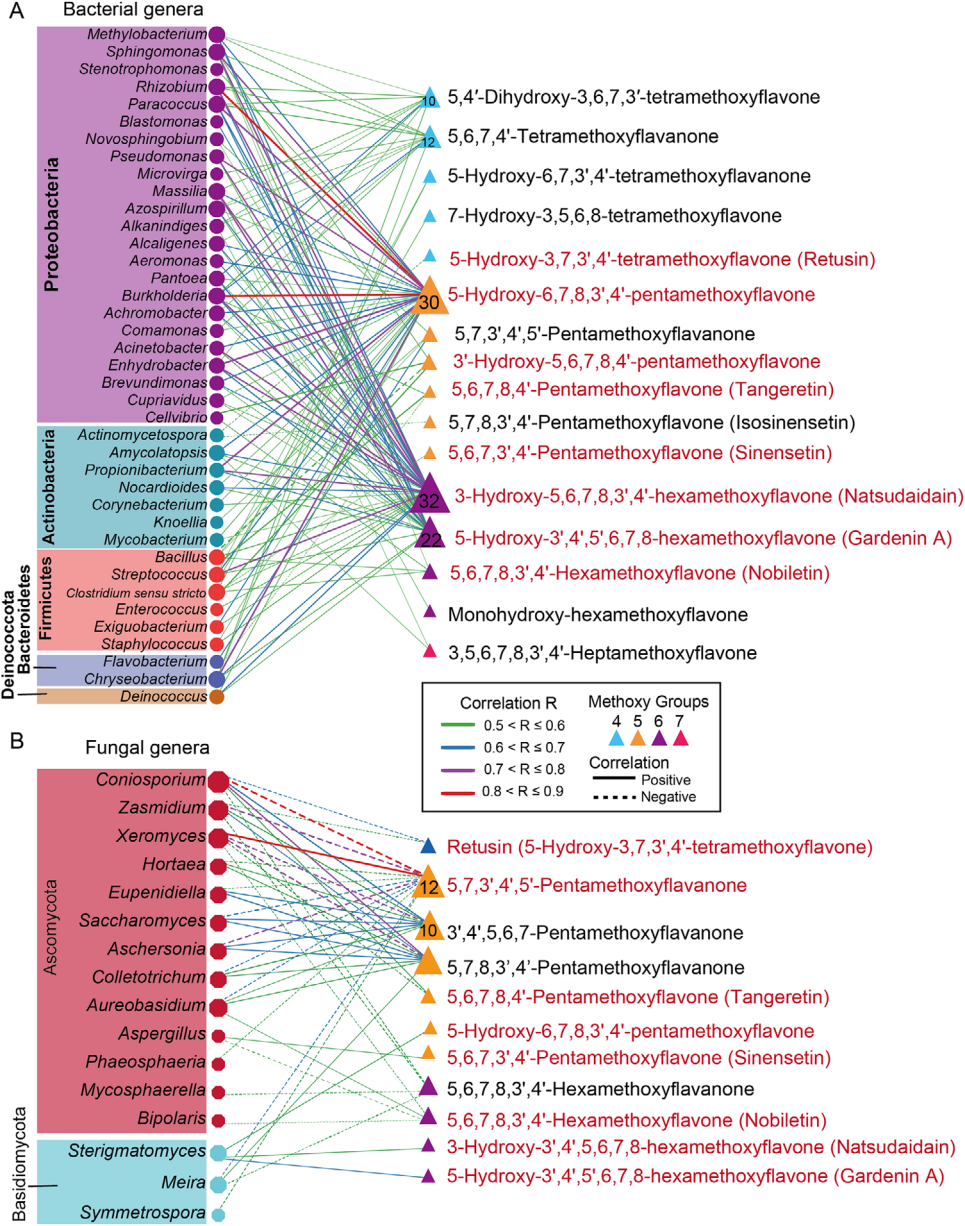
Network of associations between the bacterial composition, fungal composition, and PMFs. Edges between points are colored according to the correlation. Solid lines represent positive correlations, and dashed lines represent negative correlations. Triangles represent PMFs in PCRC. The color of triangles represents the number of methoxy groups. Circles represent bacterial genera, colored according to the phylum taxonomy information; octagons represent fungal genera, colored according to the phylum taxonomy information. The thickness of the connecting lines between points represents the absolute value of the correlation coefficient.

Within the bacterial network, notable correlations were observed between 15 PMFs and 39 bacterial genera from 5 bacterial phyla, including Proteobacteria, Actinobacteria, Firmicutes, Bacteroidetes, and Deinococota (Figure 4A). Natsudaidain, 5‐Hydroxy‐6,7,8,3',4'‐pentamethoxyflavone, and Gardenin A exhibited positive correlations with 32, 30, 22 bacterial genera. Specifically, *Bacillus* exhibited positive correlations with tangeretin, natsudaidain (3‐Hydroxy‐5,6,7,8,3',4'‐hexamethoxyflavone) 5,7,3',4',5'‐Pentamethoxyflavanone, and negative correlations with 3'‐Hydroxy‐5,6,7,8,4'‐pentamethoxyflavone (3'‐demethylnobiletin) and 5‐Hydroxy‐6,7,8,3',4'‐pentamethoxyflavone. These bacterial genera that were highly related to PMFs may contribute to the accumulation of PMFs in PCRC.

In the fungal network, significant correlations were observed between 11 PMFs and 16 fungal genera from two fungal phyla (Figure 4B). Most fungal genera exhibited negative correlations with various PMFs, suggesting they are likely involved in the degradation of flavonoids in PCRC. For example, *Xeromyces*, the most abundant fungal genus in PCRC, exhibited particularly high correlations with 5 PMFs, including 4 negative correlations and a positive correlation. *Aspergillus* exhibited a positive correlation with sinensetin and a negative correlation with nobiletin. There were 7 PMFs present in both bacterial and fungal networks, implying that these PMFs were affected by both bacteria and fungi.

### 2.4 Synthetic Communities Boost the Accumulation of PMFs in PCRC

**FIGURE 5:**
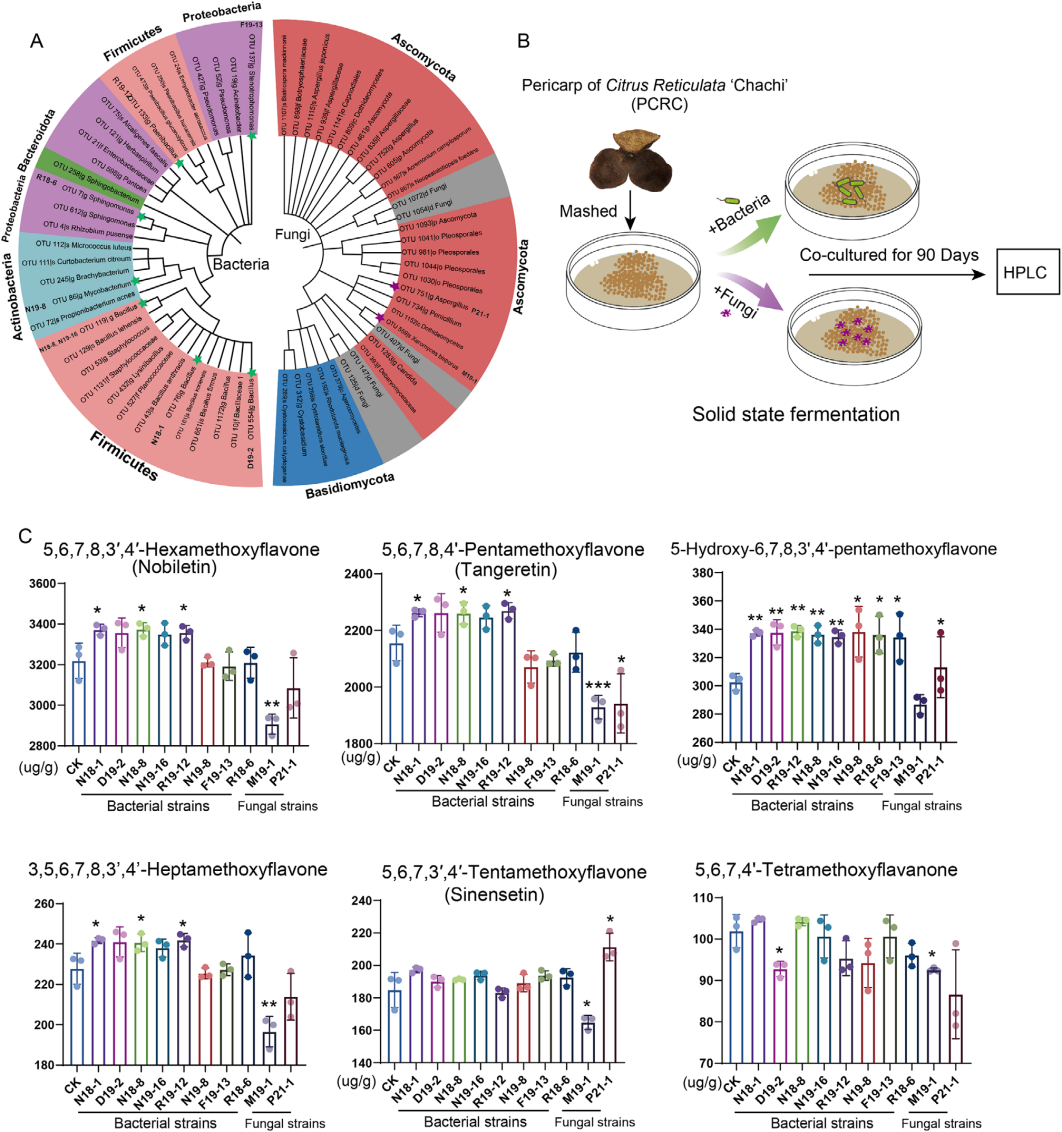
Isolation of bacteria and fungi from PCRC and fermentation experiment with selected strains. (A) Phylogenetic tree based on the sequence of OTUs matched with the isolated strains. The bacterial tree is on the left, and the fungal tree is on the right. The color of leaves represents phylum information. Strains selected for fermentation are marked by a yellow star. (B) Solid‐state fermentation of PCRC powder. PCRC was mashed to powder and co‐cultured with bacteria or fungi for 90 days. HPLC was used to detected the content of flavonoids in the PCRC powder. (C) The absolute content of six flavonoids after 90‐day fermentation with different strains (*n* = 3). Bacterial strains: N18‐1, D19‐2, N18‐8, N19‐16, R19‐12, N19‐8, F19‐13. Fungal strains: M19‐1, P21‐1. "^*^" Represent *p* < 0.05, "^**^" represent *p* < 0.01, "^***^" represent *p* < 0.001.

To investigate the impact of bacteria on the accumulation of PMFs, we isolated and identified bacterial strains from PCRC. A total of 132 bacterial strains were isolated, and 123 of them matched with 34 OTUs sequences (Figure 5A and Table S3). These bacterial strains mainly belong to 18 genera, including *Bacillus* (8 OTUs, 59 strains), *Sphingomonas* (2 OTUs, 10 strains), *Paenibacillus* (1 OTU, 11 strains), *Stenotrophomonas* (1 OTU, 1 strain), and *Mycobacterium* (1 OTU, 1 strain) (Table S3). Based on the correlation network, eight bacterial strains were selected for fermentation experiments with PCRC (Figure 5B). The absolute content of six flavonoids, including nobiletin, tangeretin, 3,5,6,7,8,3',4'‐Heptamethoxyflavone, 5‐Hydroxy‐6,7,8,3',4'‐pentamethoxyflavone, sinensetin, and 5,6,7,4'‐Tetramethoxyflavanone was quantitated. The accumulation of flavonoids was promoted by certain strains. Specifically, *Bacillus* N18‐1 increased the content of nobiletin, tangeretin, 3,5,6,7,8,3',4'‐Heptamethoxyflavone, and 5‐Hydroxy‐6,7,8,3',4'‐pentamethoxyflavone (Figure 5C). *Bacillus* D19‐2 increased the content of 5‐Hydroxy‐6,7,8,3',4'‐pentamethoxyflavone. *Bacillus* N18‐8 and *Paenibacillus* R19‐12 increased the content of nobiletin, tangeretin, 3,5,6,7,8,3',4'‐Heptamethoxyflavone, and 5‐Hydroxy‐6,7,8,3',4'‐pentamethoxyflavone. Eight strains were found to promote the accumulation of 5‐hydroxy‐6,7,8,3',4'‐pentamethoxyflavone. Three strains, including N18‐1, D19‐2, N18‐8, simultaneously boosted the accumulation of nobiletin and tangeretin. The content of sinensetin remained unchanged when fermented with any bacterial strain. These experimental results, combined with the previous correlation analysis, provide direct evidence that *Bacillus* N18‐1, N18‐8, N19‐16, *Paenibacillus* R19‐12 may increase the quality of PCRC by boosting the accumulation of PMFs during storage.

Furthermore, a total of 111 fungal strains were isolated, with 81 strains identified as matching to 33 OTU sequences from 2 phyla (Figure 5A and Table S4). Based on their relative abundance and correlation network, *Xeromyces* M19‐1 and *Aspergillus* P21‐1 were chosen for the fermentation experiment with PCRC. *Xeromyces* M19‐1 led to a decrease in content of six PMFs, including nobiletin, tangeretin, 3,5,6,7,8,3',4'‐Heptamethoxyflavone, 5‐Hydroxy‐6,7,8,3',4'‐pentamethoxyflavone, sinensetin, and 5,6,7,4'‐tetramethoxyflavanone (Figure 5C). *Aspergillus* P21‐1 boosted the accumulation of sinensetin but resulted in a decrease in nobiletin, tangeretin, 3,5,6,7,8,3',4'‐Heptamethoxyflavone, 5‐Hydroxy‐6,7,8,3',4'‐pentamethoxyflavone, and 5,6,7,4'‐Tetramethoxyflavanone (Figure 5C). These results suggest that fungi prefer to degrade PMFs in PCRC.

**FIGURE 6:**
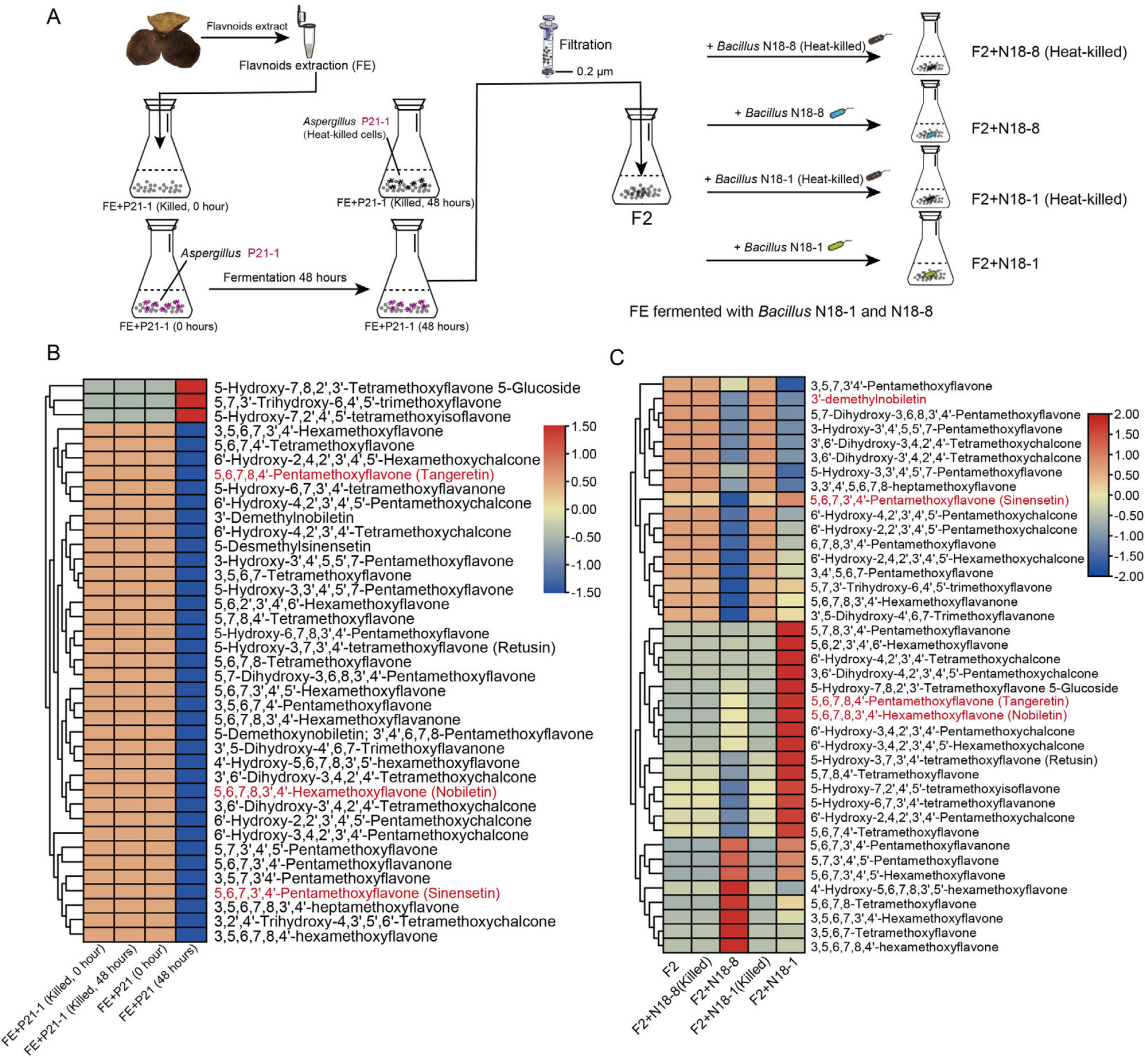
Flavonoids fermentation assays and metabolite profiles. (A) Scheme of the two‐step fermentation experiment designed to test the synergistic biotransformation of flavonoids. First, flavonoids extracted from PCRC were fermented with *Aspergillus* P21‐1 for 48 h. Heat‐killed *A. tubingensis* was used as a negative control. The resulting product (F2) was then filtered and co‐cultured with either *Bacillus subtilis* N18‐1 or N18‐8 for 12 h, with heat‐killed *Bacillus* strains serving as corresponding negative controls. The flavonoid content at each stage was analyzed using a widely targeted metabolomics. All experiments were conducted using three biological replicates. For visualization in the heatmaps, the averaged content of each PMF across the replicates was calculated, and the resulting mean value was scaled (mean‐centered and unit variance‐scaled), with colors representing relative abundance from low (blue) to high (red). (B) Heatmap of PMF profiles after the 48‐h fermentation with *A. tubingensis* P21‐1. The data show significant changes in the flavonoid profile in the presence of live P21‐1 compared to the initial state (0 h) and the heat‐killed control. FE+P21 (Killed, 0 h): Negative control; flavonoid extract immediately after inoculation with heat‐killed *A. tubingensis* P21‐1 (0 h sample). FE+P21 (Killed, 48 h): Negative control; flavonoid extract after 48 h incubation with heat‐killed *A. tubingensis* P21‐1. FE+P21 (0 h): Flavonoid extract immediately after inoculation with live *A. tubingensis* P21‐1 (0 h sample). FE+P21 (48 h): Flavonoid extract after 48 h fermentation with live *A. tubingensis* P21‐1. (C) Heatmap of PMF profiles after the subsequent 12‐h co‐culture with *B. subtilis* strains. The results demonstrate further transformations of the flavonoid profile by live N18‐1 and N18‐8 compared to the F2 starting material and the heat‐killed controls. F2: The starting material (filtered product from the live *A. tubingensis* fermentation). F2+N18‐1 / F2+N18‐8: F2 after 12 h co‐culture with live *B. subtilis* N18‐1 or N18‐8. F2+N18‐1(Killed) / F2+N18‐8(Killed): Negative controls; F2 after 12 h incubation with the respective heat‐killed *B. subtilis* strains.

To further explore the effect of bacteria and fungi in the fermentation of PMFs, we extracted flavonoids from PCRC and fermented them with *Aspergillus* P21‐1, *Bacillus* N18‐1, and *Bacillus* N18‐8 (Figure 6A), respectively. The fermentation of *Aspergillus* P21‐1 with flavonoids extract (FE) showed that the content of 36 PMFs decreased after fermentation, such as nobiletin, tangeretin, 3,5,6,7,8,3',4'‐Heptamethoxyflavone, 5‐Hydroxy‐6,7,8,3',4'‐pentamethoxyflavone, sinensetin, and 5,6,7,4'‐Tetramethoxyflavanone (Figures 6B and S12). Only three PMFs increased, including 5‐Hydroxy‐7,8,2',3'‐tetramethoxyflavone 5‐glucoside, 5,7,3'‐Trihydroxy‐6,4',5'‐trimethoxyflavone, 5‐Hydroxy‐7,2',4',5'‐tetramethoxyflavone (Figure 6B). While the content of flavonoids did not change significantly between FE+P21‐1 (Killed, 0 h) and FE+P21‐1 (Killed, 48 h), implying that killed cells of Aspergillus P21‐1 can't transform flavonoids. (Figure 6B). After fermentation for 48 h, the product was filtered and added into NB medium. *Bacillus* N18‐1 or N18‐8 was co‐cultured with the filtrated product for 12 h, respectively (Figure 6A). As a result, the content of 9 PMFs increased, and 32 PMFs decreased in the product of fermented *Bacillus* N18‐8 (Figure 6C). The increasing PMFs included nobiletin, tangeretin, 3,5,6,7,8,4'‐Hexamethoxyflavone, 3,5,6,7‐Tetramethoxyflavone, 3,5,6,7,3',4'‐Hexamethoxyflavone. Notably, fermentation with *Bacillus* N18‐1 showed a greater number of increased PMFs than N18‐8, with 20 PMFs upregulated, including tangeretin, nobiletin, and sinensetin. In contrast, 3'‐Hydroxy‐5,6,7,8,4'‐pentamethoxyflavone (3'‐demethylnobiletin), a precursor of tangeretin, sharply decreased after fermentation (Figure 6C). The flavonoids content showed no changes between the heat‐killed groups and F2 group. These results implied that *Bacillus* N18‐1 may further transform the fermentation products of P21‐1 to synthesize tangeretin.

**FIGURE 7:**
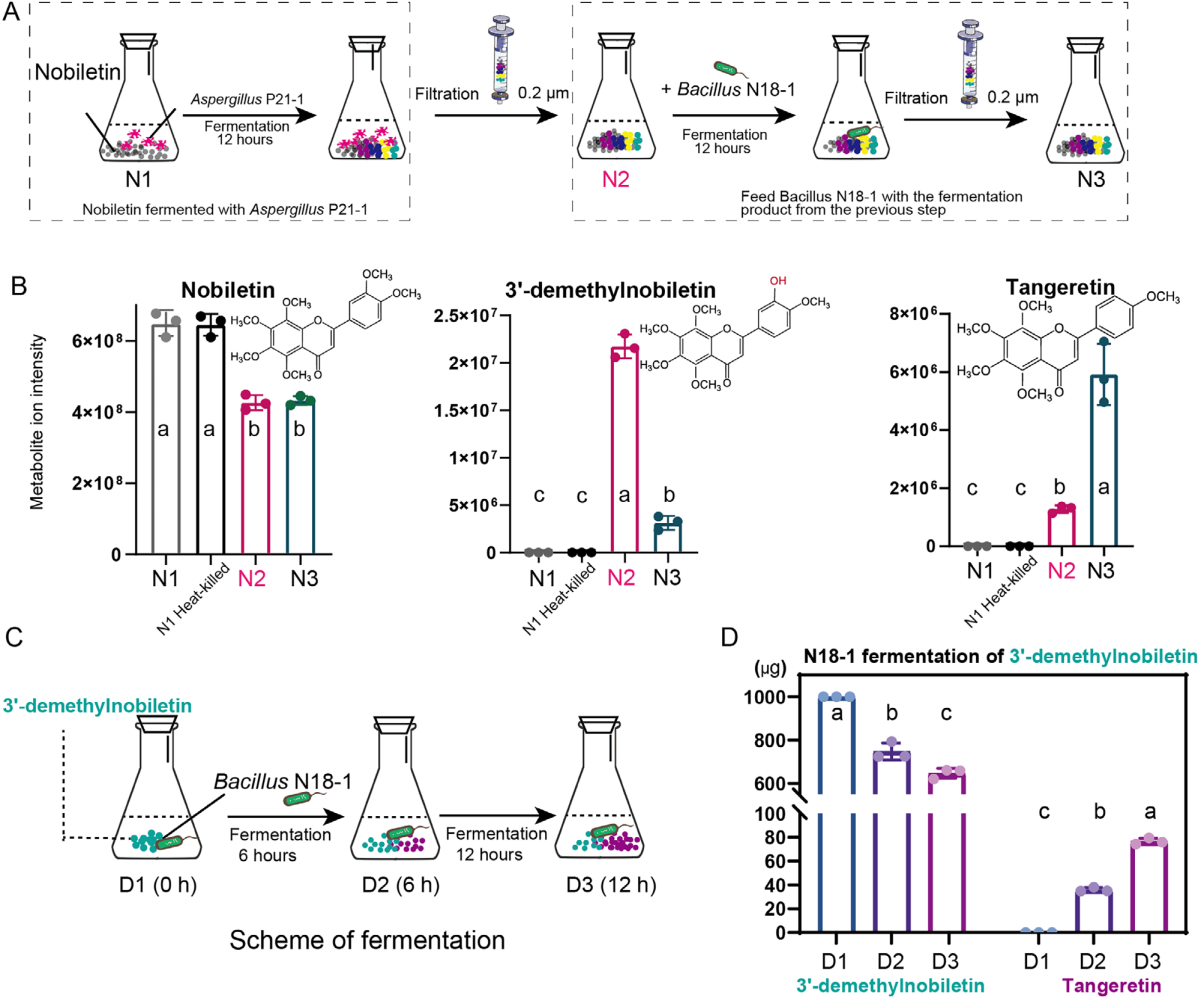
In vitro validation of the synergistic two‐step biotransformation of nobiletin to tangeretin. (A) Schematic of the sequential fermentation experiment. First, nobiletin was fermented with *Aspergillus tubingensis* P21‐1 for 12 h. Second, the sterile‐filtered product from Step 1 was subsequently fermented with *Bacillus subtilis* N18‐1 for another 12 h. (B) Relative abundance of nobiletin, 3'‐demethylnobiletin, and tangeretin at each stage of the sequential fermentation. Flavonoid levels were determined using widely targeted metabolomics. N1: The initial state (0 h). N1 Heat‐killed: A negative control showing no change after 12 h with heat‐killed *A. tubingensis*. N2: After 12 h with *A. tubingensis*, nobiletin is partially consumed, and a large amount of 3'‐demethylnobiletin is produced. N3: After the subsequent 12 h with *B. subtilis*, 3'‐demethylnobiletin is consumed, and a large amount of tangeretin is produced. (C) Schematic of the direct fermentation experiment to confirm the second step of the pathway, where *B. subtilis* N18‐1 was fed directly with the intermediate, 3'‐demethylnobiletin. Samples were taken at 0 h (D1), 6 h (D2), and 12 h (D3). (D) Absolute quantification (µg) of 3'‐demethylnobiletin and tangeretin during the direct fermentation shown in (C). The results confirm that *B. subtilis* N18‐1 directly consumes 3'‐demethylnobiletin and produces tangeretin over a 12‐h period. Bar graphs in (B) and (D) represent the mean ± SD of three biological replicates. Different lowercase letters (a–c) above the bars indicate statistically significant differences (*p* < 0.05) as determined by one‐way ANOVA with Tukey's posthoc test.

Based on our previous results, we hypothesized that *Aspergillus* P21‐1 can transform certain PMFs to form O‐demethylate PMFs through O‐demethylation, and *Bacillus* N81‐1 further converts the O‐demethylate PMFs to other PMFs. To verify this hypothesis, we fed *Aspergillus* P21‐1 with nobiletin for 12 h, then filtered the product to feed N18‐1 (Figure 7A). The content of nobiletin decreased, while 3'‐Hydroxy‐5,6,7,8,4'‐pentamethoxyflavone (3'‐demethylnobiletin) and tangeretin increased after feeding *Aspergillus* P21‐1 with nobiletin (Figure 7B). Subsequently, we observed that the content of tangeretin increased and 3'‐demethylnobiletin sharply decreased in the fermentation of N18‐1(Figure 7B), while the content of nobiletin remained unchanged (Figure 7B). These results further supported that P21‐1 probably demethylated nobiletin to form 3'‐demethylnobiletin, then *Bacillus* N18‐1 further transformed the 3'‐demethylnobiletin to form tangeretin.

To further verify this hypothesis, *Bacillus* N18‐1 was fed with 3'‐demethylnobiletin (Figure 7D). The content of 3'‐demethylnobiletin decreased at 6 h and 12 h fermentation, while the content of tangeretin increased simultaneously (Figure 7E). These results support that 3'‐demethylnobiletin can be transformed to tangeretin by *Bacillus* N18‐1.

To approach the interaction between *A. tubingensis* P21‐1 and *B. subtilis* N18‐1 in aging PCRC, reported antimicrobial substances genes affecting each other to survive were tested. The relative expression level of two surfactin biosynthesis genes (*srfAA* and *srfAC*) and one cephalosporin‐C deacetylase gene was downregulated in co‐culture compared with mono‐culture (Figure S12). This result indicates a symbiotic relationship between *A. tubingensis* P21‐1 and *B. subtilis* N18‐1 to maintain flavonoid conversion through cross‐feeding between them.

### 2.5 Gene Identified and Validation of Catalytic Activity in Yeast

**FIGURE 8:**
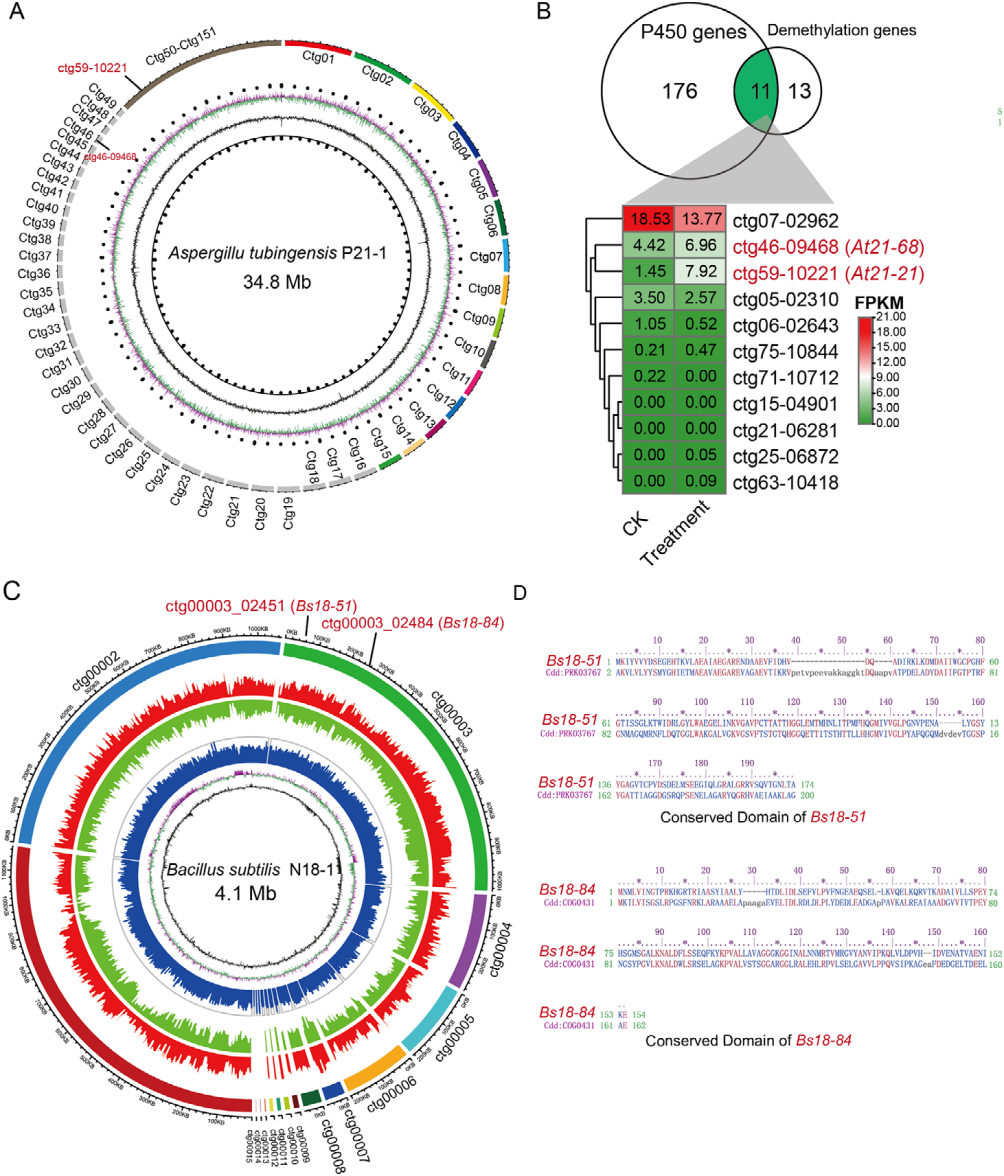
Genome and gene identification of *Aspergillus tubingensis* P21‐1 and *Bacillus subtilis* N18‐1. (A) Genome features of *Aspergillus tubingensis* P21‐1. The inner circle shows the shows GC skew, and the blue bar represents the mean coverage of each read. The outer circle shows 151 contigs sorted by size, and genes involved in the flavonoid synthesis pathway are marked on the contigs. (B) Expression of 11 demethylation genes in CK and treatment groups. The Venn diagram represents the number of cytochromes P450 oxygenase and demethylation genes. Raw FPKM values are shown. (C) Genome features of *Bacillus* N18‐1. The inner circle shows GC skew, and the blue bar represents the mean coverage of each read. Red and green bars represent the coverage of reads1 and reads2, respectively. The outer circle shows 103 contigs sorted by size, and genes involved in the flavonoid synthesis pathway are marked on the contigs. (D) Conserved domains of two candidate flavone reductases. The orange box represents the domains of NAD(P)H‐dependent *FMN* reductase.

To further identify the genes involved in the transformation of PMSs, the genome of *Aspergillus* P21‐1 was sequenced and assembled, resulting in a 34.8 Mb genome (Figure 8A). *Aspergillus* P21‐1 was identified as *Aspergillus tubingensis* based on 99% similarity of ITS with *Aspergillus tubingensis*. In total, 11,115 genes were predicted and annotated. Among them, 19 genes were identified with demethylation function, and 187 genes were identified as cytochrome P450 oxygenase, which belong to 9 subfamilies (Figure S14A). Eleven genes are involved in the intersection between demethylase and cytochrome P450 oxygenase (Figure 8B). Transcriptomic sequencing and analysis of *Aspergillus tubingensis* P21‐1 (ck) and P21‐1 fed with Nobiletin (treatment) showed that two genes (*ctg46‐09468* abbreviated as *At21‐68*, *ctg59‐10221* abbreviated as *At21‐21*) were upregulated in the treatment groups (Figure 8B), quantitative PCR (qPCR) confirmed this result (Figure S13). Molecular docking analysis of enzymes encoded by these two genes interacting with nobiletin yielded docking score −4.34 and −3.36, respectively (Figure S14B,C). These results support the potential role of these enzymes in the O‐demethylation of PMFs.

The genome of *Bacillus* N18‐1 was 4.14 Mb (Figure 8C) and annotated as *Bacillus subtilis*. A total of 4241 coding genes were identified. Among them, two genes (*ctg00003_02451* abbreviated as *Bs18‐51*, *ctg00003_02484* abbreviated as *Bs18‐84*) were identified as the flavone reductase (*FLR*), which has potential to transform 3'‐demethylnobiletin to tangeretin (Figure 8D). Further molecular docking of Bs18‐51 and Bs18‐84 with 3'‐demethylnobiletin exhibited dock score −2.63 and −2.14, respectively (Figure S14D,E), indicating that Bs18‐51 and Bs18‐84 exhibit the potential to transform 3'‐demethylnobiletin to tangeretin.

**FIGURE 9:**
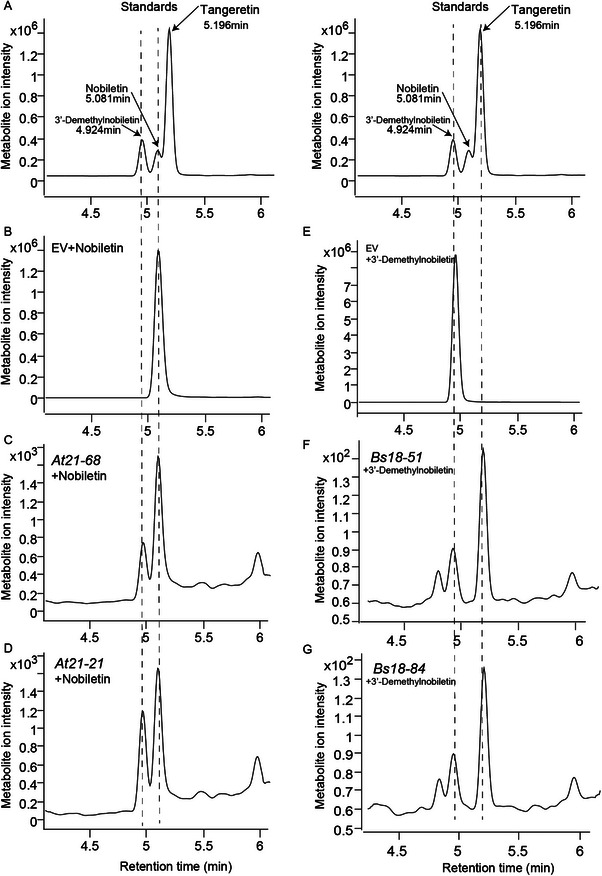
Functional validation of candidate genes from *A. tubingensis* and B. *subtilis* by heterologous expression in Saccharomyces cerevisiae. Representative UPLC‐MS chromatograms demonstrate the catalytic activity of the four identified enzymes. (A) Chromatogram of an authentic standard mixture showing the retention times for 3'‐demethylnobiletin (4.924 min), nobiletin (5.081 min), and tangeretin (5.196 min). (B–D) Conversion of nobiletin. Yeast expressing an empty vector (EV) shows no conversion of nobiletin (B). In contrast, yeast expressing *A. tubingensis* genes At21‐68 (C) and At21‐21 (D) successfully converts nobiletin into 3'‐demethylnobiletin, as indicated by the appearance of a new peak at 4.924 min. (E–G) Conversion of 3'‐demethylnobiletin. Yeast expressing an empty vector (EV) shows no conversion of 3'‐demethylnobiletin (E). However, yeast expressing *B. subtilis* genes Bs18‐51 (F) and Bs18‐84 (G) converts 3'‐demethylnobiletin into tangeretin, evidenced by the appearance of a new peak at 5.196 min. These results confirm the specific enzymatic functions required for the two‐step biotransformation pathway.

To further validate the function of *At21‐68*, *At21‐21*, *Bs18‐51*, and *Bs18‐84*. We utilized the pRS416 vector for expressing to validate catalytic activity of these four genes in yeast. The ultra performance liquid chromatography tandem mass spectrometry (UPLC–MS/MS) was used to quantify the content of 3'‐demethylnobiletin, nobiletin, and tangeretin (Figure S16). As a result, At21‐68 and At21‐21 showed the demethylase activity and catalyzed the conversion of nobiletin to 3'‐demethylnobiletin, while Bs18‐51 and Bs18‐84 catalyze the conversion of 3'‐demethylnobiletin to tangeretin (Figure 9). The results of the catalytic activity verification assays indicate that these four genes are directly involved in the conversion of nobiletin to tangeretin.

In summary, fungi and bacteria synergistically reprogram PMFs biosynthesis by fungal *At21‐68*, *At21‐21* and bacterial *Bs18‐51*, *Bs18‐84*, resulting in rich and diverse PMFs in PCRC.

## 3 Discussion

Aging of medicinal materials is a traditional practice in Chinese medicine, involving the storage and maintenance of medicinal materials through specific methods, with subsequent utilization after a period of aging [19]. Aging medicines enhance clinical efficacy by reducing toxicities and side effects, generating new bioactive substances, and enhancing bioavailability [26, 27, 28]. For hundreds of years, the mature pericarp of *Citrus reticulata* 'Chachi'' (PCRC) has been used as an aging traditional Chinese medicinal material [6, 29]. Recent reports consistently emphasize the beneficial effects of prolonged storage of PCRC, revealing that the contents of polyphenols, flavonoids, and particularly PMFs undergo changes during the aging process [5, 16, 23, 30].

To decipher the aging mechanism, we utilized UPLC‐MS/MS and widely targeted metabolomics analysis to comprehensively explore the content and composition of flavonoids in PCRC throughout a storage period ranging from 0 (fresh peel) to 19 years groups. Most of the PCRC samples from the same year exhibited similar flavonoid contents. However, a small amount of variation was also observed, which may be caused by differences in agronomic or environmental factors between samples. The metabolite content changed dramatically after 2 years of aging. Specifically, the total content of 22 PMFs increased from 2 years of storage. Since PMFs possess a wide range of bioactivities [31, 32], these findings provided evidence that PCRC aged for more than two years has better clinical efficacy. In addition, we also found that hesperetin‐7‐O‐glucoside, hesperetin‐5,7‐di‐O‐glucoside, and gallocatechin were detected only in aged PCRC, not in fresh PCRC. Previous studies reported that hesperetin‐7‐O‐glucoside and gallocatechin exhibited various biological activities, including anti‐inflammatory, anti‐viral, and anti‐tumor properties [33, 34, 35, 36]. Thus, these three flavonoids can be used as marker components to discriminate between newly harvested and aged PCRC. These results reveal for the first time that aging not only increases the content of the biologically active component PMFs, but also produces new bioactive flavonoids.

Previous studies indicated that environmental factors, specifically humidity and temperature, elevate the levels of PMFs and affect the composition of coexisting microbial populations during storage [37, 38]. In our study, the PCRC were collected from the same orchards and stored in the same humidity and temperature, indicating that the changes in flavonoid content may be caused by other factors. Further systematic research should focus on how environmental factors influence the composition of components in PCRC by affecting changes in microbial composition, thereby revealing the intricate relationships and patterns among the environment, microorganisms, and metabolites. Previous studies reported that the chemical compositions and contents of traditional Chinese medicine were changed after microbial fermentation [39, 40]. For example, the bacterial community is closely associated with specific compositions in essential oils and phenolics, which contribute to the flavor and quality of PCRC [23]. Generally, bacteria and fungi are competitors in the ecosystem, and the competition for resources dominates these interactions [41]. Our studies revealed that the abundance of dominant bacterial genera such as *Methylobacterium*, *Pseudomonas*, *Rhizobium*, and *Bacillus* was initially low from CK to GCP2, then rapidly increased from GCP3 to GCP5, and subsequently decreased sharply in GCP6. In contrast, the dominant fungal genus *Saccharomyces* and *Aspergillus* sharply decreased from GCP6 and maintained low abundance throughout the 19‐year storage period. Bacteria and fungi always coexist with each other in soils and form complex interactions that are crucial for their survival, adaptation, establishment, maintenance, and functions [42, 43, 44]. Among these interaction types, competition for resources dominates these interactions in soils [45, 46, 47]. PCRC, as “soil” for its attached bacteria and fungi, the limitation of carbon and energy sources induced competition between bacteria and fungi. Certain volatile compounds, produced by bacteria, emerged as inhibiting fungal growth [48]. As a result, the increase in the bacterial Shannon index may have caused the decrease in the fungal Shannon index. Additionally, microbial community clustering showed a pattern similar to the clustering of metabolites in PCRC, indicating that the diversity of bacteria and fungi changed as the content of flavonoids changed during PCRC aging.

We observed a significant discrepancy in the abundance of endophytic microbial communities detected by next‐generation sequencing (NGS) compared with traditional culture‐dependent methods. Consistent with previous findings, this divergence can be primarily attributed to inherent limitations of surface sterilization approaches, suboptimal incubation conditions, and insufficient duration for slow‐growing or fastidious taxa to proliferate [49, 50, 51]. These factors collectively lead to substantial differences in both the diversity and relative abundance of microbial species captured by culture‐dependent vs. culture‐independent techniques. To address these limitations, more advanced techniques, such as scanning electron microscopy of sterilized tissue and qPCR of surface vs. interior DNA, should be employed to verify the thoroughness of surface sterilization, thereby ensuring the isolation of truly endophytic microorganisms.

The dominant bacteria and fungi in PCRC may affect the content of flavonoids in PCRC [24, 30]. Indeed, a recent report suggested that two fungi, *Monascusanka* and *Saccharomyces cerevisiae*, can increase free flavonoid content during fermentation with PCRC [52]. Here, we provide novel evidence that bacteria and fungi synergistically reprogram the metabolites of flavonoids in PCRC during storage. Three *Bacillus* species, including *Bacillus subtilis* N18‐1, D19‐2, and N18‐8 boosted the accumulation of PMFs, while fungal genera, including *Aspergillus tubingensis* P21‐1 and *Xeromyces* M19‐1 decreased PMFs in both the fermentation of the PCRC powder and flavonoid extracts. The mechanism of synergy between bacteria and fungi is as intricate as microbial dark matter [24]. Recent progress indicates that the biodegradation of bisphenol A is facilitated by a synergistic interaction involving cross‐feeding of three different microorganism species [53]. Metabolite exchange keeps microbial communities ecologically stable, playing a crucial role in fermented food [54, 55]. Synergy between microorganisms allows the products of some microorganisms to be used as substrates for others to synthesize new products [56, 57]. Taken together, our research reveals that within complex microbial networks, bacteria and fungi act synergistically, not only maintaining microbial balance, but also participating together in the reshaping of metabolites for the synthesis of PMFs during the PCRC aging process.

Further feeding experiments confirmed that *Aspergillus* P21‐1 degraded nobiletin to produce 3'‐demethylnobiletin, and subsequently *Bacillus* N18‐1 converted 3'‐demethylnobiletin into tangeretin. *Aspergillus* is capable of various biochemical reactions, including O‐demethylation, hydroxylation, dehydroxylation, deglycosylation, dehydrogenation, hydrogenation, cyclization, and reduction [58]. Buisson et al. found that *Aspergillus niger* ATCC 9142 transformed tangeretin to 4′‐hydroxytangeretin by O‐demethylation [59]. In our research, two O‐demethylation genes (*At21‐68*, *At21‐21*) were identified in *Aspergillus* P21‐1, and their biological function was subsequently verified in yeast. Both enzymes were shown to reduce nobiletin levels by O‐demethylating the methyl group at the 3' position of nobiletin to produce 3'‐demethylnobiletin. Additionally, two flavone reductase genes (*Bs18‐51*, *Bs18‐84*) of PMFs were identified in *Bacillus* N18‐1. Their biological function was also verified in yeast, demonstrating that both enzymes can transform 3'‐demethylnobiletin to tangeretin.

Overall, metabolomics and microbial amplicon sequencing data revealed that the *Aspergillus* abundance was negative with the content of nobiletin and *Bacillus* abundance was positive with the content of tangeretin from 0–19 years of storage PCRC. Further fermentation assays and enzyme catalytic activity verification assays of *Aspergillus* P21‐1 and *Bacillus* N18‐1 confirmed that *Aspergillus* P21‐1 reduces nobiletin to produce 3'‐demethylhesperidin by At21‐68 and At21‐21 of the demethylase activity, and *Bacillus* N18‐1 transforms 3'‐demethylhesperidin to produce tangeretin by Bs18‐51 and Bs18‐84 of reductase activity. Previous studies have demonstrated that the interaction between *Aspergillus* and *Bacillus* constitutes a mutualistic, actively adaptive process, whereby the two microorganisms establish peaceful coexistence through downregulating the synthesis of antimicrobial substances (surfactin in *Bacillus* and cephalosporin in *Aspergillus*) [60, 61]. Our findings provide direct molecular evidence for this mechanism in the context of PCRC aging. We observed that the biosynthesis genes for these antimicrobial substances were significantly downregulated in co‐culture compared to mono‐culture. This mutual downregulation indicates a symbiotic relationship that creates a stable microenvironment conducive to metabolite exchange. It is within this cooperative context that the sequential biotransformation of flavonoids occurs: a symbiotic relationship is achieved and maintained, allowing for the efficient cross‐feeding of 3'‐demethylnobiletin from *A. tubingensis* to *B. subtilis*. Taken together, our metabolomics data, functional validation experiments, and gene expression analysis fully reveal that the coordinated action of bacteria and fungi during the aging process of PCRC promotes the degradation of nobiletin into 3'‐demethylnobiletin, which is subsequently further transformed into tangeretin.

Our findings offer compelling evidence for improving the medicinal efficacy of PCRC after aging by microbial communities in PMFs synthesis within PCRC. This achievement provides new highlights for facilitating the transformation of the bioactive ingredients in traditional Chinese medicine or food through the management of bacteria and fungi. Our results also provide a new insight into genetic engineering and further transform medicinal ingredients to accelerate the production of high‐quality medicinal products.

## 4 Material and methods

### 4.1 Plant Material

The fruits of *C. reticulata* 'Chachi' were collected from Shuangshui Town, Xinhui District, Jiangmen City, Guangdong Province, China, in November of 2001, 2002, 2004, 2009, 2012, 2014, 2015, 2016, 2017, 2018, 2019, and 2020. The authenticity of all samples was confirmed by Professor Wei Liu.

Upon harvest, any stains on the fruit's surface were washed off with clean water. The pericarp was then peeled off, dried in sunlight, and stored in sealed bags for aging. The PCRC was stored in a cool and dry place and periodically exposed to sunlight several times throughout the year for drying. Three biological replicates were included in each group. More detailed information about the samples can be found in Table 1 and Supplementary Table S6.

**TABLE 1:** PCRC Samples Information.

Groups	Harvest time	Aging period	Common name	Origin
CK	2020	0	Freshly harvested pericarps	Shuangshui Town, Xinhui District, Jiangmen City, Guangdong Province, China
GCP1	2019	1	1‐year‐old pericarps	
GCP2	2018	2	2‐year‐old pericarps	
GCP3	2017	3	3‐year‐old pericarps	
GCP4	2016	4	4‐year‐old pericarps	
GCP5	2015	5	5‐year‐old pericarps	
GCP6	2014	6	6‐year‐old pericarps	
GCP8	2012	8	8‐year‐old pericarps	
GCP11	2009	11	11‐year‐old pericarps	
GCP16	2004	16	16‐year‐old pericarps	
GCP18	2002	18	18‐year‐old pericarps	
GCP19	2001	19	19‐year‐old pericarps	

### 4.2 Sample Preparation

For flavonoids metabolomic sequencing, PCRC samples were freeze‐dried and subsequently crushed using a mixer mill (MM 400, Retsch) for 1.5 min at 30 Hz. 100 mg of powder from each sample was weighed and extracted using 1.0 mL of 70% aqueous methanol. The mixtures were stored overnight at 4°C and vortexed three times to ensure complete extraction. After centrifugation at 10 000 g for 10 min, the extracts were filtered (SCAA‐104, 0.22 µm pore size) before undergoing UPLC‐MS/MS analysis.

For 16S rRNA gene and ITS amplicon sequencing, the PCRC samples were cut into small pieces measuring 1 cm in length and width. These fragments were placed in 15‐mL tubes containing 10 mL of sterile PBS‐S buffer. Subsequently, the samples were washed for 20 min on a shaking platform at 25 rotations per second to effectively remove microorganisms tightly adhered to the surface of the pericarps. After washing, the pericarp pieces were sterilized by exposure to 75% alcohol for 1 min, followed by thorough rinsing with sterile water five times. To verify the effectiveness of this surface sterilization protocol, the final rinse water used to wash the PCRC samples was plated onto nutrient broth (NB) agar plates. These plates were then incubated at 37°C for 7 days. The absence of significant microbial growth on these plates confirmed that our method effectively removed the vast majority of surface‐adhering microbes. Finally, the clean pericarps were collected to extract the DNA of the endophytic microbiome using the CTAB method.

### 4.3 Metabolic Profiling

The PCRC samples were freeze‐dried by a vacuum freeze‐dryer (Scientz‐100F). The freeze‐dried sample was crushed, and 100 mg of lyophilized powder was dissolved in 1.2 mL 70% methanol solution. The mixture was vortexed for 30 s every 30 min for 6 times, and then placed in a refrigerator at 4°C overnight. The sample extracts were analyzed using an LC–ESI–MS/MS system. Linear ion trap (LIT) and triple quadrupole (QQQ) scans were acquired on a triple quadrupole‐linear ion trap mass spectrometer (Q TRAP), API 4500 Q TRAP LC/MS/MS System, equipped with an ESI Turbo Ion‐Spray interface operating in a positive ion mode. The HPLC chromatograms diagram of all samples and QC samples are shown in supplementary Figures S17–S19. Variable importance in projection (VIP) values were extracted from the OPLS‐DA results, which were generated using R package MetaboAnalystR [62]. Significantly regulated metabolites between groups were determined by VIP ≥ 1 and absolute Log_2_FC (fold change) ≥ 1.

### 4.4 Amplicon Sequencing and Data Analyzing

The V4 region of the bacterial 16S rRNA gene was amplified with primers 515F (5'‐GTGCCAGCMGCCGCGGTAA‐3'), 806R (5'‐GGACTACHVGGGTWTCTA AT‐3'), while the fungal internal transcribed spacer (ITS) gene was amplified with ITS1(5'‐AGAAGTCGTAACAAGGTTTCCGTAGG‐3'), ITS4 (5'‐TCCTCCGCTTATTGAT‐ATGCTTAA‐3'). The amplified products were sequenced on the BGISEQ platform. Raw reads were filtered by SOAPnuke (version 2.1.7) to remove adaptor sequences and trim low‐quality reads [63]. A total of 50 000 reads were subsampled from each sample and dereplicated by VSEARCH (version 2.15.2) with the commands –derep_fulllength [64]. Chimeras were removed with the command –uchime_denovo. Operational Taxonomic Units (OTUs) were clustered by the command –id 0.97 –cluster_fastcat. The original reads were assigned back to their OTUs using USEARCH (version 10.0) [65]. OTUs and sequences defined as unknown, chloroplast, mitochondria, or plants were removed. Representative sequences of each OTUs were aligned against the RDP (version11.4) and UNITE (version 10.0) database, respectively [66, 67].

### 4.5 Construction of Correlation Network Between Microorganisms and Metabolites

Spearman correlation coefficients between microorganisms and metabolites were calculated using the cor.test function from the “stats” package in R. To account for multiple comparisons, the Benjamini–Hochberg method was applied to correct the *p‐*value obtained from the Spearman correlation analysis [68]. The filtering criteria were set as FDR‐corrected *p* value ≤ 0.05 and an absolute correlation coefficient (R) ≥ 0.5. The filtered results were then visualized using Cytoscape (version 3.7.0) [69], displaying the correlation coefficients (R) and corresponding *p‐*value.

### 4.6 Bacterial Strains Isolation and Identification

For the isolation of bacterial strains, the aging PCRC samples were ground in a mortar. Afterwards, a 1 g sample was diluted in 10 mL of PBS buffer and thoroughly mixed on a shaking platform for 10 min at 25 revolutions per second. The supernatants were serially diluted from 10^−1^ to 10^−7^, with the 10^−4^ and 10^−6^ dilutions selected for plating. These dilutions were spread and cultivated on Nutrient Agar, Flavobacterium Medium, Reasoner's 2A agar (R2A), Dextran agar medium, and Cellulose Congo Red medium for 7 days at 30°C. Following purification through three consecutive platings on solidified media, a total of 132 bacterial clones were obtained. The full‐length bacterial 16S rRNA gene was amplified and sequenced with primers 27F (5'‐AGAGTTTGATCMTGGCTCAG‐3'), 806R (5'‐GGTTACCTTGTTACGACTT‐3'). The bacterial strains were identified by comparing the sequences to our bacterial OTUs sequences, with a threshold of 97% gene similarity.

To isolate fungal strains, the PCRC samples were cut into small cubes and cultivated on Malt Extract, Yeast Extract 50% Glucose Agar medium for 14 days at 28°C. Following purification through three consecutive platings on solidified media, 111 fungal clones were obtained. The fungal ITS was amplified and sequenced with primers ITS1 (5'‐AGAAGTCGTAACAAGGTTTCCGTAGG‐3'), ITS4 (5'‐TCCTCCGCTTATTGATATGCTTAA‐3'). The fungal strains were identified based on their best match with our fungal OTUs sequences, with a gene similarity greater than 97%.

### 4.7 Solid‐State Fermentation (SSF) of PCRC

Strains were resuscitated and cultured on Nutrient Agar medium. After confirming the purity and activity of the strain, they were subcultured to the second generation. Inoculum cultivation of bacteria was carried out in a 250‐mL flask containing 50 mL of the corresponding seed medium and incubated at 30°C and 180 rpm for 30 h. The cultures were centrifuged to remove the culture medium, and the precipitates were collected and washed 2 to 3 times with sterile deionized water to remove the liquid and obtain fresh bacterial cells.

The 2‐year PCRC was mashed, and large particles were filtered out. Five grams of PCRC powder were evenly spread in the petri dish. The strain (4% w/w) was mixed evenly with the PCRC powder and placed in the petri dish. The petri dishes were placed in an incubator at 25°C and 75% humidity. After 90 days of fermentation, the total flavonoids of the PCRC powder were extracted by 70% methanol as mentioned above.

To determine the content of six flavonoids (including nobiletin, tangeretin, 3,5,6,7,8,3',4'‐Heptamethoxyflavone, 5‐Hydroxy‐6,7,8,3',4'‐pentamethoxyflavone, sinensetin, and 5,6,7,4'‐Tetramethoxyflavanone), the standards of six flavonoids were used to build a calibration curve at an Agilent 1260 HPLC system equipped with a vacuum degasser, a G1316A quadruple pump, a G1315C diode array detector (DAD) and a G1367E autosampler. The mobile phase A was acetonitrile, and mobile phase B was 0.1% formic acid at a flow rate of 0.8 mL/min. The column temperature was set at 40°C with an injection volume of 5 µL and a UV detection wavelength of 340 nm and 280 nm. The samples were run with the following gradient program: 0–10 min, 10%‐15% acetonitrile; 10–16 min, 15%‐20% acetonitrile; 16–19 min, 20%‐90% acetonitrile; 22–27 min, 10% acetonitrile.

### 4.8 Liquid‐State Fermentation (LSF) of Flavonoids Extraction

Total flavonoids of PCRC were extracted and configured to 0.1 g/mL as a stock solution. Strains were resuscitated and cultured in potato dextrose broth (PDB). After confirming the purity and activity of each strain, they were subcultured to the second generation. Inoculum cultivation was carried out in a 250‐mL flask containing 100 mL of the corresponding seed medium and incubated at 30°C and 180 rpm for 60 h. 2 mL flavonoid stock solution was added into PDB to ferment for 2 days. After fermentation, the total flavonoids in the fermentation broth were extracted by 70% methanol as mentioned above. Heat‐killed cells were used as negative controls. The heat‐killed temperature was 95°C and the duration was 10 min. The content of flavonoids in liquid‐state fermentation assays was analyzed using an LC–ESI–MS/MS system as previously described.

### 4.9 Liquid‐State Fermentation (LSF) of Nobiletin and 3’‐Demethylnobiletin

Initially, *Aspergillus* P21‐1 cultivated on potato dextrose agar (PDA), was inoculated into potato dextrose broth (PDB) culture medium, followed by the addition of 1 mL of 0.1 mg/L nobiletin for co‐fermentation over 24 h. The resulting product was then filtered through a 0.2‐micron microporous membrane to obtain sterile samples. Subsequently, 1.0 mL of the filtered product was co‐fermented with *Bacillus* N18‐1 for 6 and 12 h. After filtration, all products were analyzed using the LC–ESI–MS/MS system as previously described.

For 3'‐demethylnobiletin fermentation assays, *Bacillus* N18‐1 was inoculated into NB culture medium, then 1 mg 3'‐demethylnobiletin was added into the medium. After co‐fermentation for 6, 12 h, products were filtered and analyzed using the Agilent 6470 triple quadrupole LC/MS with 1290 front end. The standards of 3'‐demethylnobiletin and tangeretin were used to build a calibration curve to determine absolute quantification of 3'‐demethylnobiletin and tangeretin in the samples.

### 4.10 Genome and Transcriptome Sequencing Analysis of *Aspergillus P21‐1* and *Bacillus* N18‐1

Total DNA of *Aspergillus* P21‐1 and *Bacillus* N18‐1 was extracted and sequenced on the Illumina platform, obtaining 2.66Gb and 1.62Gb of PE150 cleaned data, respectively. The genome was assembled using SPAdes (version 3.5.0) [70], and the assembled contig was further completed using GapFiller (version 1.11) [71]. The resulting sequences were corrected using PrInSeS‐G (version 1.0.0) [72]. The assembled genomes were subjected to gene element prediction using the NCBI Prokaryotic Genome Annotation Pipeline [73]. Predicted genes were functionally annotated using BLAST by aligning them against CDD, KOG, COG, NR, NT, InterPro, Swissprot, and TrEMBL databases. Total RNA was extracted and sequenced on the Illumina platform. The cleaned data were mapped to the genome by Bowtie2 (version 2.4.1) [74]. Gene expression was estimated by featureCounts (version 2.0.1) [75].

### 4.11 Flavone Biosynthesis Genes Identified in *Aspergillus* P21‐1 and *Bacillus* N18‐1

Demethylation genes in *Aspergillus P21‐1* were identified by annotation against the NR databases. All genes annotated with demethylation function were identified, and those with FPKM >1 were selected. For flavone reductase (*FLR*) genes in *Bacillus* N18‐1, the NADPH‐dependent FMN reductase domain (PF03358) was downloaded from InterPro, and the genes were identified by HMMER [76]. Ten genes were initially identified and were then narrowed down to two genes after blasting to a reported *FLR* gene [77].

### 4.12 Molecular Docking

The optimal binding sites between At21‐68, At21‐21, and nobiletin were predicted by the molecular docking method (AutoDock version 4.2) [78]. The protein structures of Bs18‐51 and Bs18‐84 were predicted using AlphaFold (https://alphafoldserver.com/) [79]. The structure of nobiletin was obtained from PubChem (https://pubchem.ncbi.nlm.nih.gov/compound/) [80]. Proteins were treated by removing water molecules and adding Gastieger charges and polar hydrogen atoms before docking. The docking site of nobiletin on At21‐68 and At21‐21 was defined at the active site with the grid box size of 124 × 108 × 125 and 126 × 120 × 113, respectively. The optimal docking model from AutoDock 4.2 was visualized using PyMol 2.4.1 (https://pymol.org). A Similar method was used to perform the optimal binding site between Bs18‐51, Bs18‐84, and 3'‐demethylnobiletin. The docking site of nobiletin on At21‐68 and At21‐21 was defined at the active site with the grid box size of 120 × 126 × 115 and 124 × 125 × 126, respectively.

### 4.13 Validation of Gene Catalytic Activity in Yeast

The functional validation of gene *At21‐68*, *At21‐21*, *Bs18‐51*, *Bs18‐84* refers to our previous studies [81]. In brief, the sequences of four genes were ligated with the TDH3 promoter and ADH1 terminator sequences before insertion into the pRS416 vector, respectively. The constructed pRS416‐gene vector was then transformed into the yeast strain T300. As a control group, empty pRS416 was simultaneously integrated into the T300 strain. Yeast is initially cultured in 250 mL conical flask containing 20 mL YPD medium at 30°C and 200 rpm for 8 h. Subsequently, 1 mL of the culture was transferred into 250 mL conical flask containing 20 mL of SD medium. Add nobiletin or 3'‐demethylnobiletin to the culture as needed, with a final concentration of 0.1 mg/L for each compound. The fermentation medium was incubated at 30°C and 200 rpm for 8 h.

## Author Contributions

H.W. conceived and designed the project; J.M.S., H.Y.B., L.D.L., S.S.Z., M.B., and H.J.H., collected and processed the samples. J.M.S. and H.Y.B. performed bioinformatics analysis. J.M.S., H.Y.B., L.D.L. and S.S.Z. performed the isolation and identification of microorganisms. J.M.S., L.D. L. and S.S.Z. performed the solid‐state and liquid‐state fermentation. J.M.S., H.Y.B., L.D.L., S.S.Z., and M.B. interpreted the data and wrote the original draft of the manuscript. H.W., H.L., Y.Y.W., and X.X.L. helped with editing the manuscript. All authors discussed the results, and H.W. and Y.S. helped revise the manuscript. The authors read and approved the final manuscript.

## Conflicts of Interest

The authors declare no conflicts of interest.

## Supporting information




**Supporting File 1**: advs74292‐sup‐0002‐SuppMat.docx.

**Supporting File 2**: advs74292‐sup‐0001‐Tables.xlsx.

## Data Availability

The raw data generated in this study were submitted to the NCBI BioProject database (https://www.ncbi.nlm.nih.gov/bioproject/) under accession number PRJNA986684 and are publicly available as of the date of publication. All original code has been deposited at https://github.com/sjm042/GCP‐Microbiome and is publicly available as of the date of publication. Requests for further information and resources should be directed to and will be fulfilled by the lead contact, Hong Wu (wh@scau.edu.cn).
