# Supplementary material for: Bacteria and Fungi Synergistically Reprogram Flavonoid Metabolites in the Pericarp of Citrus Reticulata 'Chachi' During Storage
Source: Adv Sci (Weinh). 2026 Feb 9;13(22):e00267. doi: 10.1002/advs.202500267 (PMC13088269; doi:10.1002/advs.202500267)

**Supplemental figures with corresponding titles and legends:**

**Figure S1. Heatmap of metabolites abundance in all groups.** Clustering analysis of flavonoid metabolites in 12 groups of PCRC. The accumulation level of metabolites is indicated by color, ranging from red (high) to green (low).

**Figure S2. Volcano plots of differential metabolites.** Note: Each point in the volcano plot represents a metabolite. The x-axis represents the logarithmic value of the quantitative fold change between two sample groups. The y-axis represents the VIP value. Red points indicate down-regulated differential metabolites, blue points indicate up-regulated differential metabolites, and grey points indicate insignificant changes.

**Figure S3. Metabolites co-upregulated in all 11 groups of PCRC.** The x-axis represents the group names.

**Figure S4. Flavonoids exclusively identified in aging PCRC.**

**Figure S5. Bacterial Species Accumulation Curves**

**Figure S6. Bacterial composition at the phylum level.**

**Figure S7. Mean abundance of the top 20 bacterial genera**

**Figure S8. Correlation between PMFs and bacterial O-methyltransferases.** The heatmap shows the OMTs abundance scale. Green triangles represent PMFs in PCRC, and purple circles represent bacterial OMTs. Lines connecting PMFs and OMTs represent the correlation coefficient R value.

**Figure S9. Fungal Species Accumulation Curves**

**Figure S10. Fungal composition at the phylum level.**

**Figure S11. Fungal composition at the genus level.** Only the top 20 fungal genera by relative abundance are shown.

**Figure S12. Bar plots showing absolute quantities of key PMFs**

**Figure S13. Relative expression levels of four surfactin biosynthesis genes and one cephalosporin-C deacetylase gene.** ‘*’ represent P value < 0.01; ‘ns’ represent not significant.

**Figure S14. qPCR validation of RNA-seq data for keys genes**

**Figure S15. Phylogenetic relationships of cytochrome P450 oxygenase and molecular docking of four key genes.**

(A) Phylogenetic relationships of cytochrome P450 oxygenase. The inner heatmap represents gene expression levels of cytochrome P450 oxygenase. The blue bars represent gene length. The outer ring represents the superfamily of cytochrome P450 oxygenase. Green represents low expression, and red represents high expression.

(B-C) Molecular docking of At21-68, At21-21 and nobiletin.

(D-E) Molecular docking of Bs18-51, Bs18-84 with 3'-demethylnobiletin.

**Figure S16. Multiple Reaction Monitoring (MRM) chromatograms of keys PMFs**. A, B, C represent MRM information of nobiletin, 3’-demethylnobiletin, tangeretin, respectively. In each panel, extracted ion chromatogram and product ion spectrum were showed.

**Figure S17. Total ion chromatograms of mix samples**

**Figure S18. Multiple reaction monitoring chromatograms of multimodal maps (Samples)**

**Figure S19. Multiple reaction monitoring detection of multimodal maps (QC)**

**Figure S1**


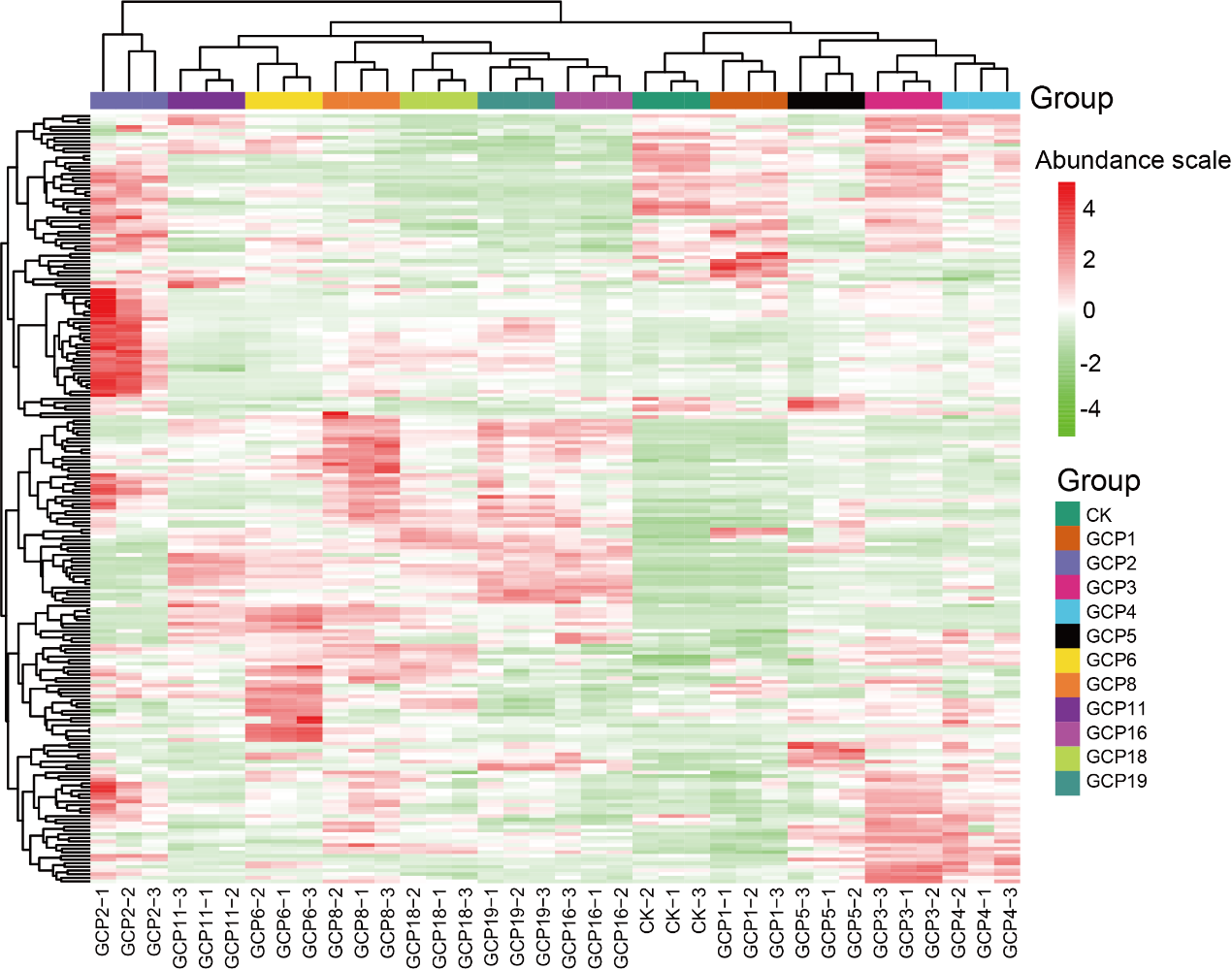


**Figure S2**


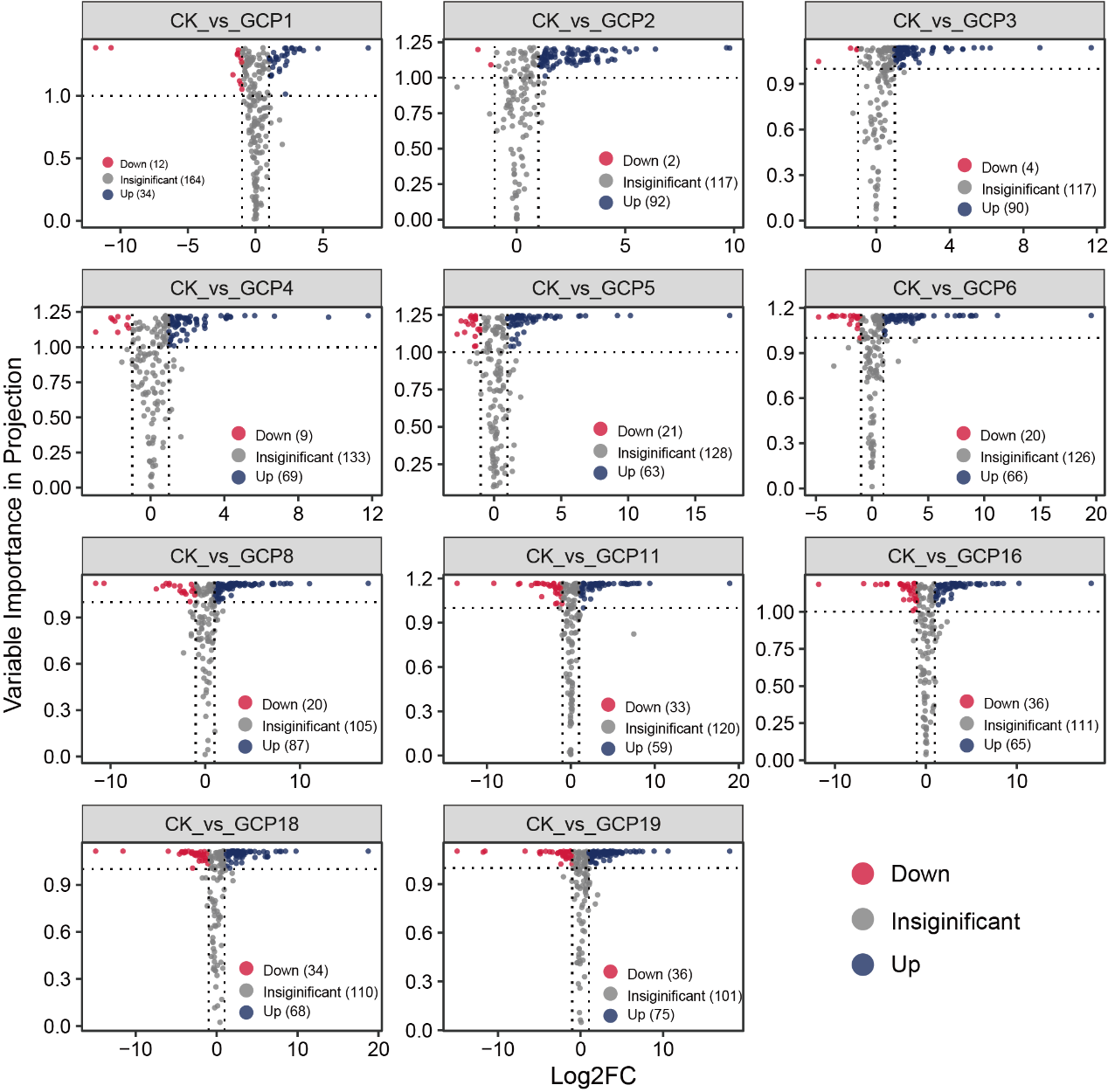


**Figure S3**


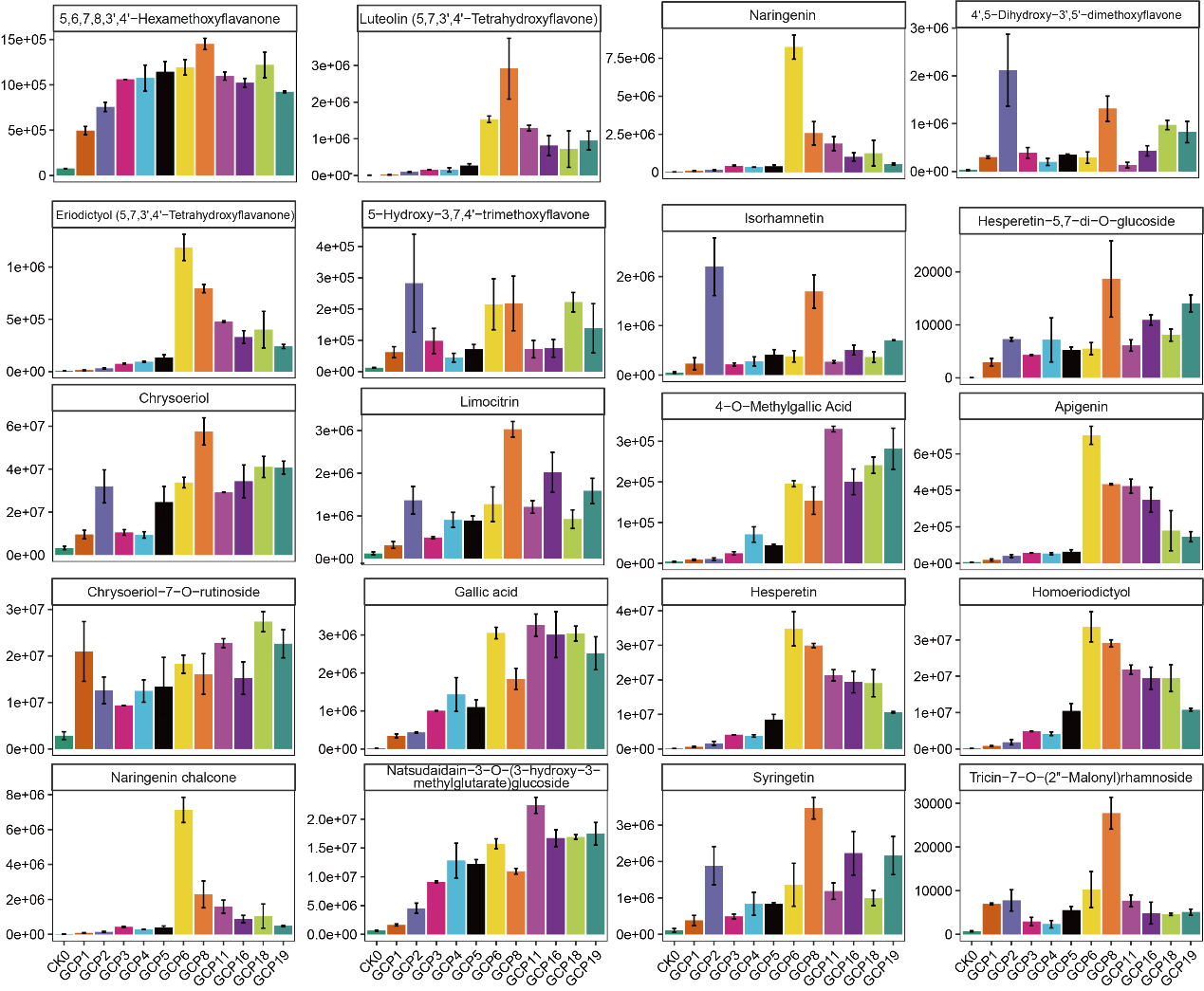


**Figure S4**


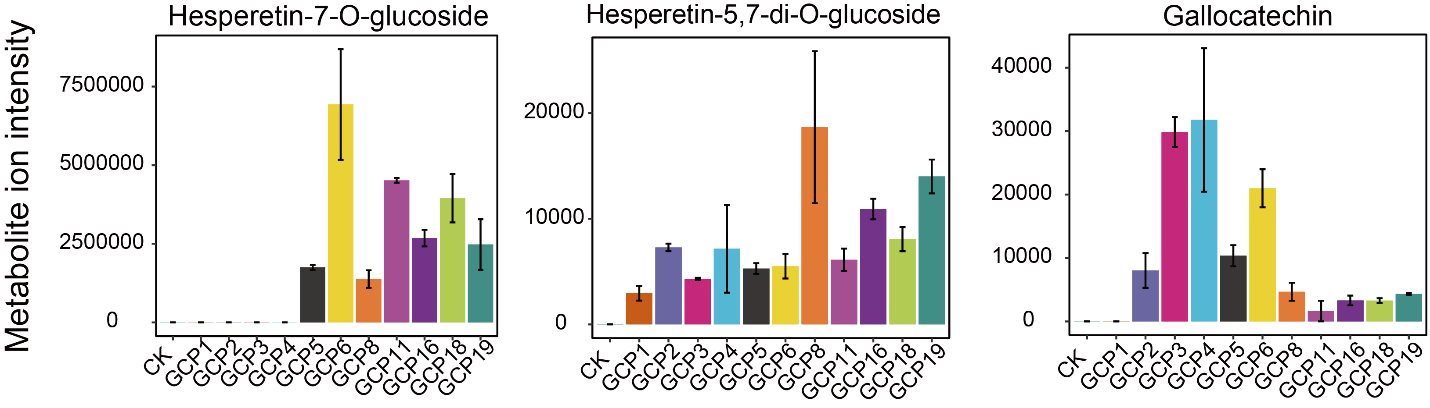


**Figure S5**


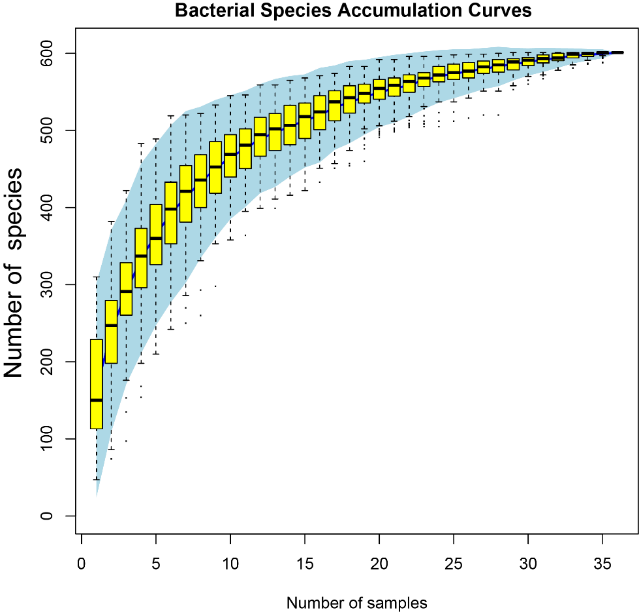


**Figure S6**


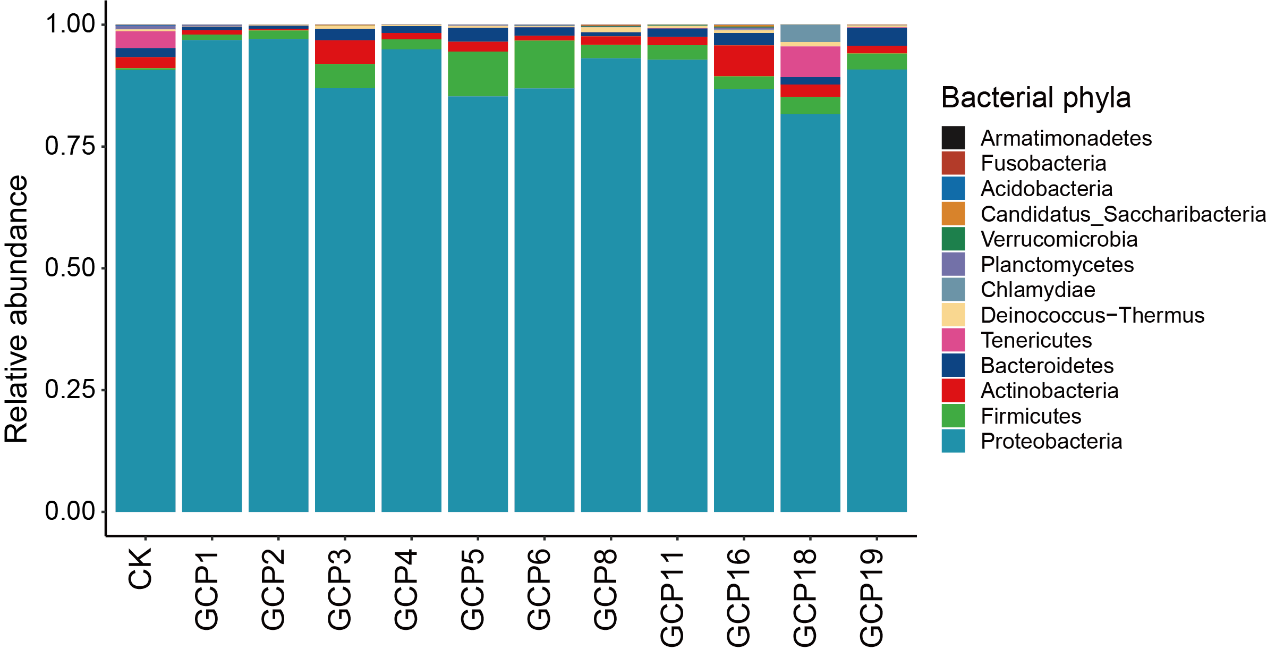


**Figure S7**


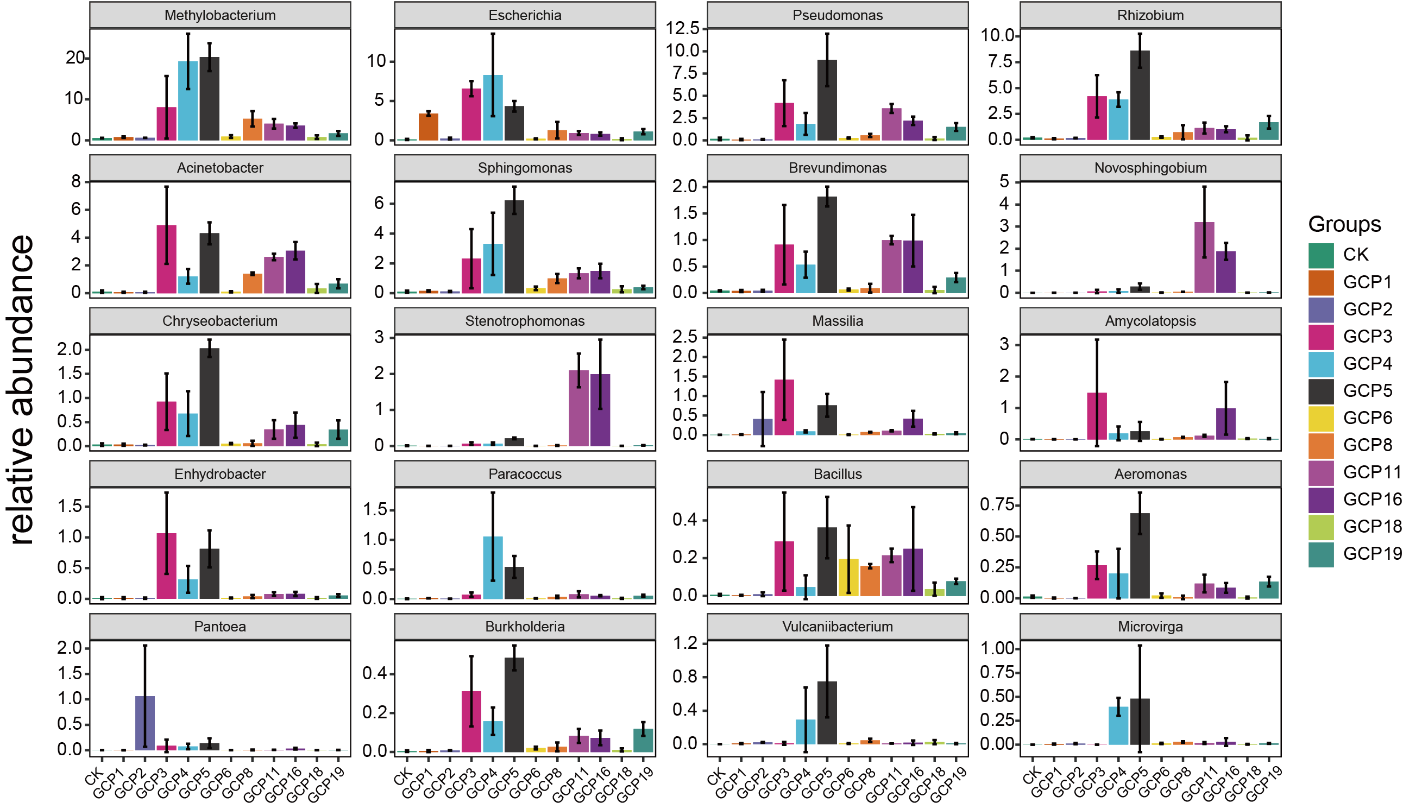


**Figure S8**


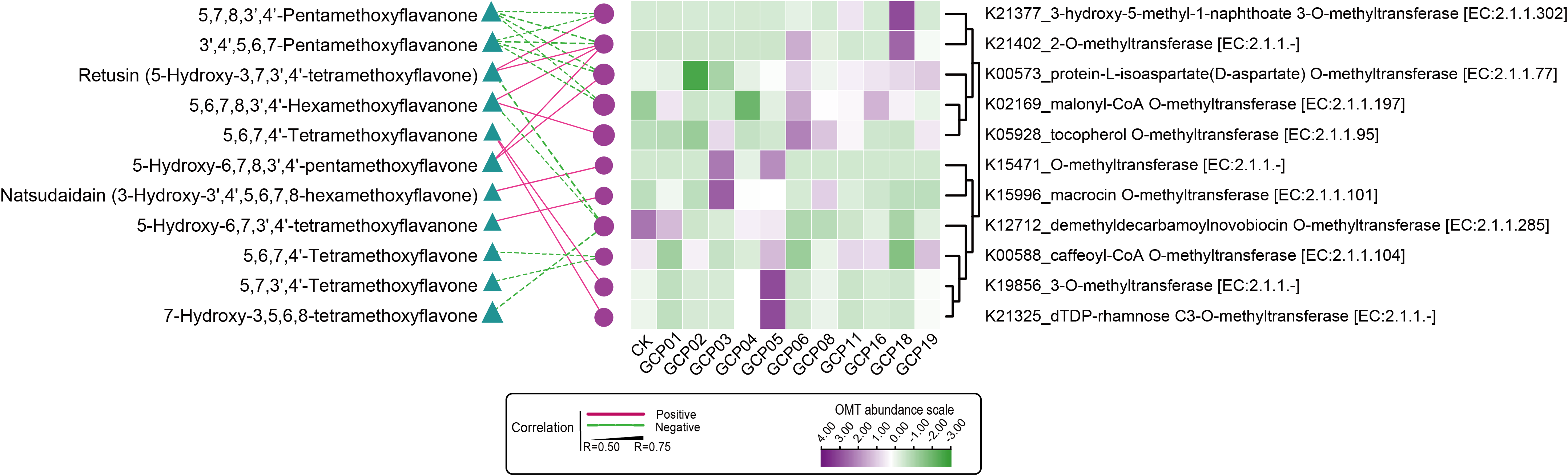


**Figure S9**


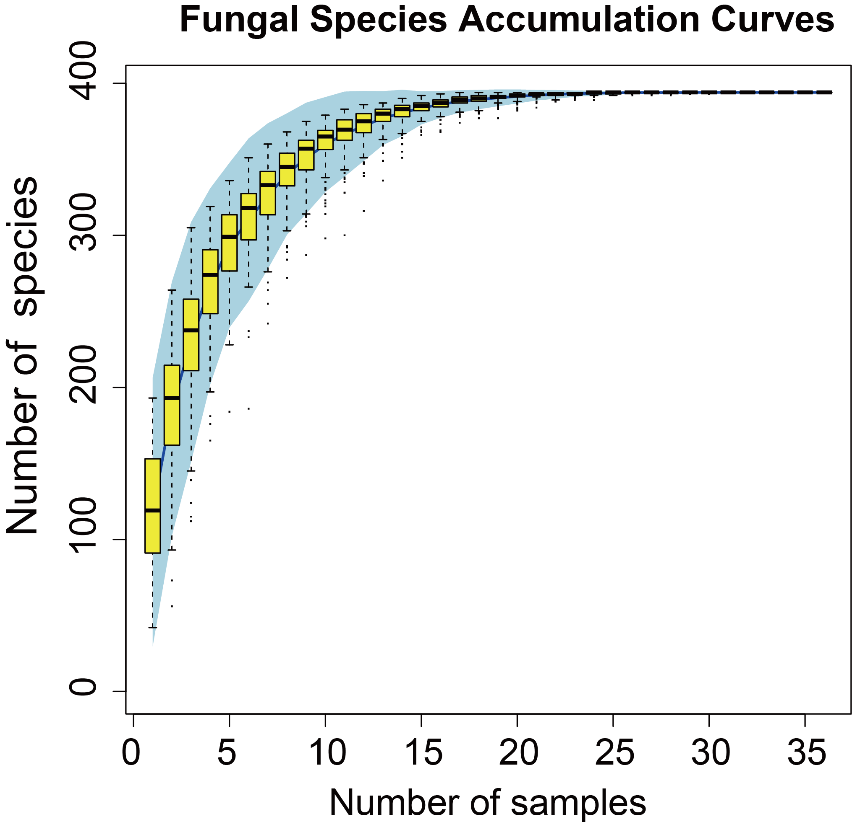


**Figure S10**


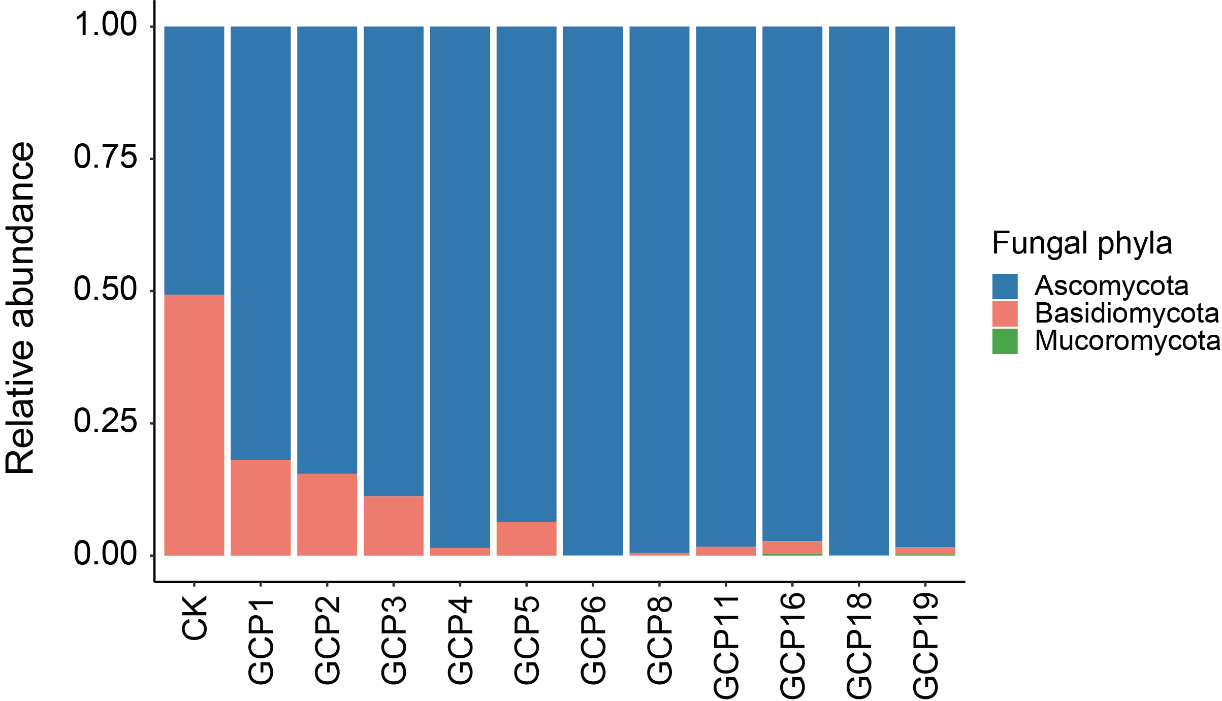


**Figure S11**


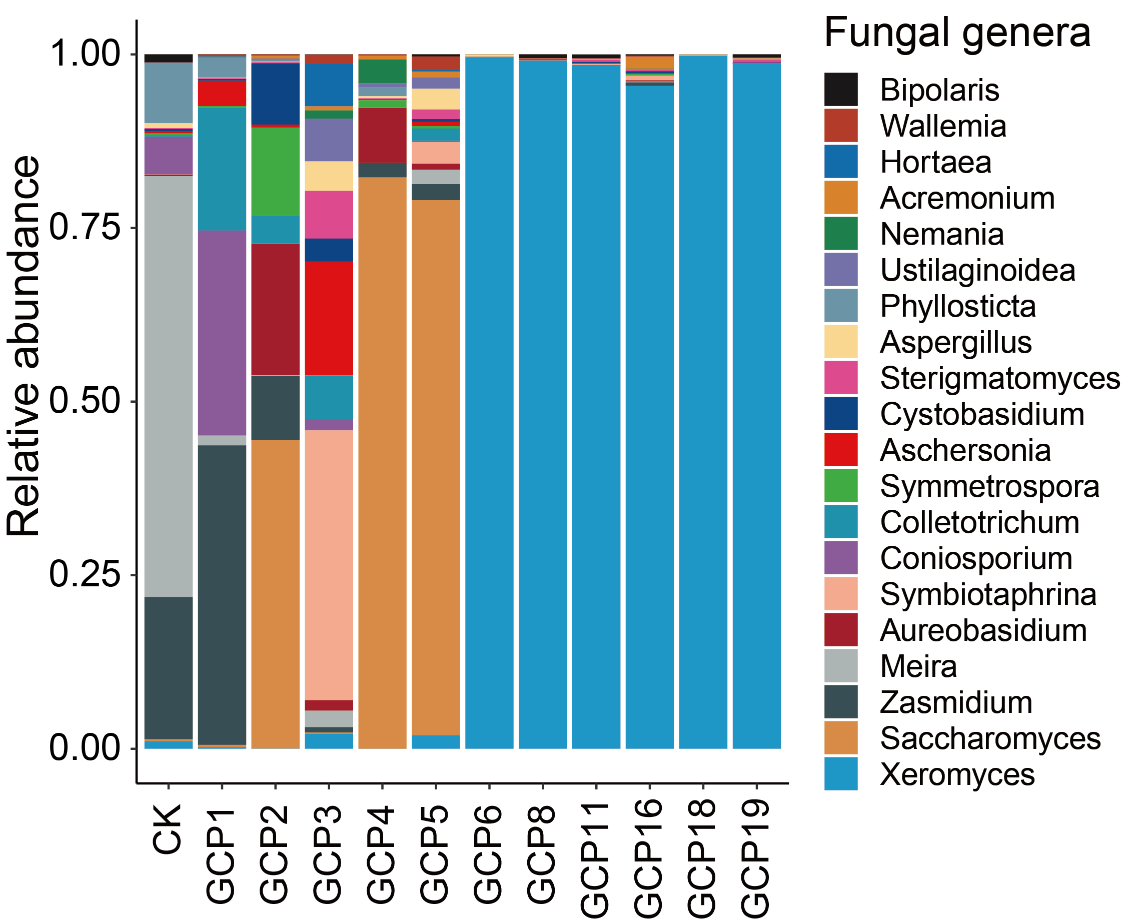


**Figure S12**


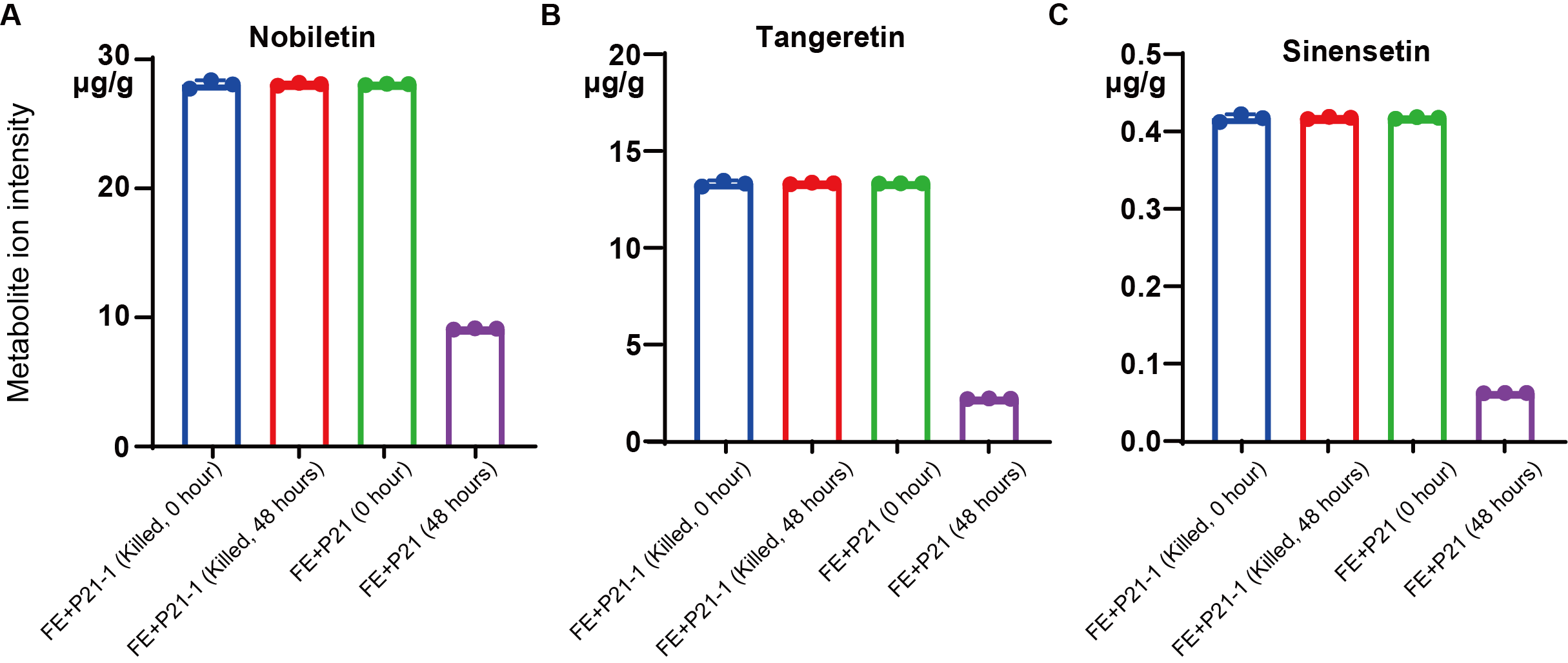


**Figure S13**


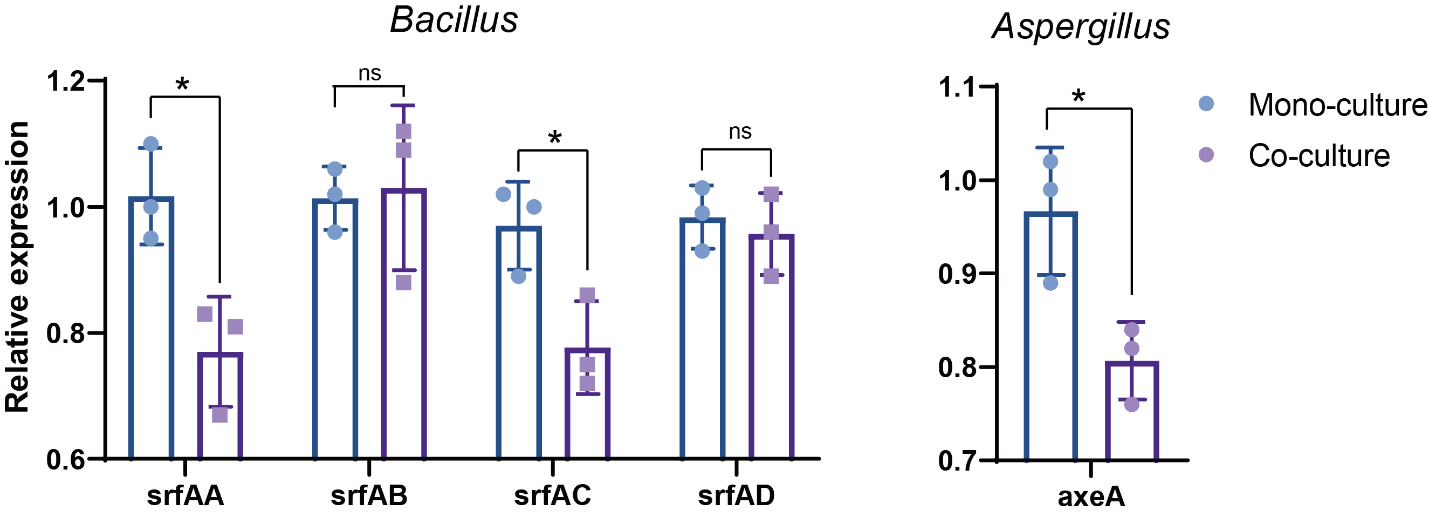


**Figure S14**


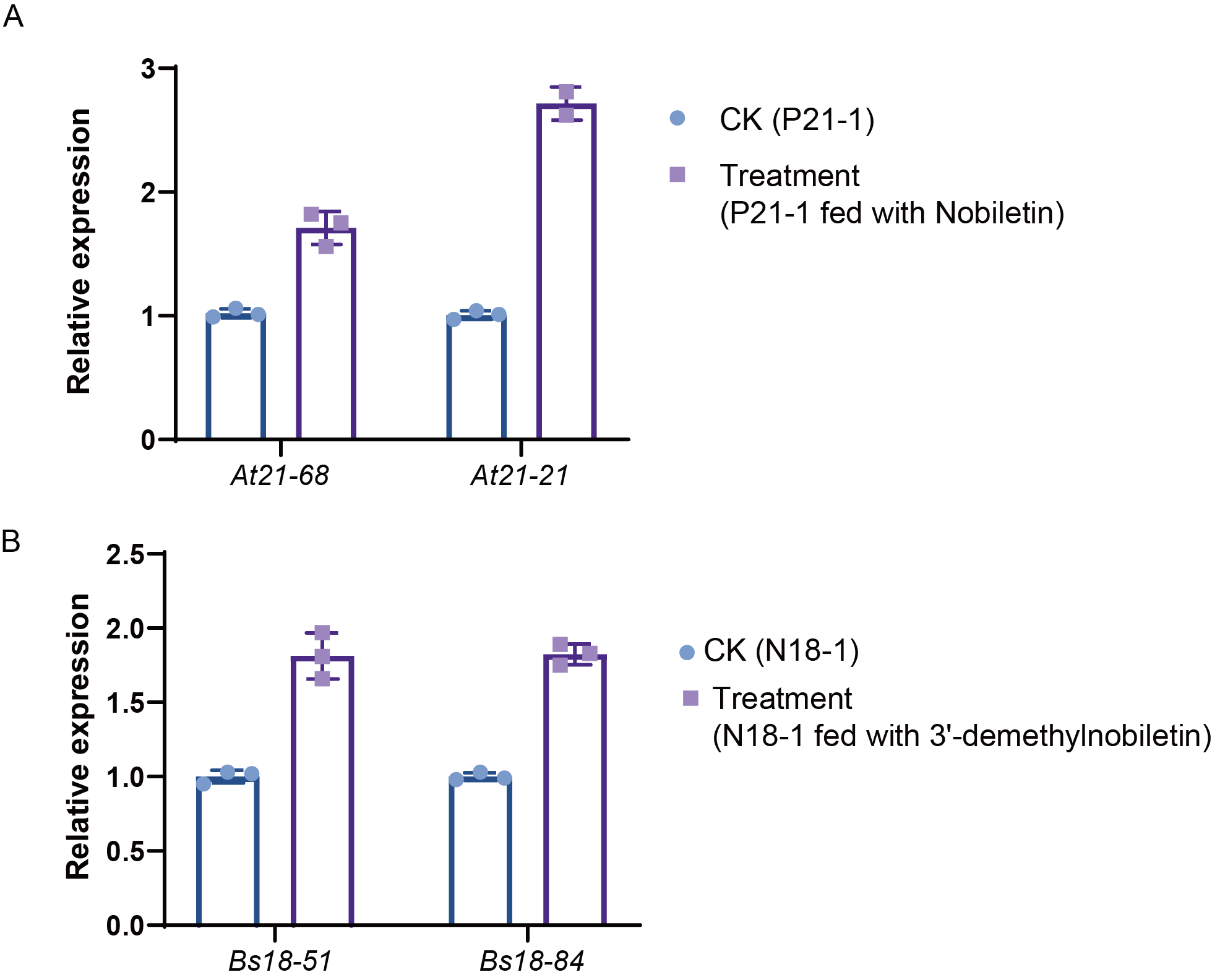


**Figure S15**


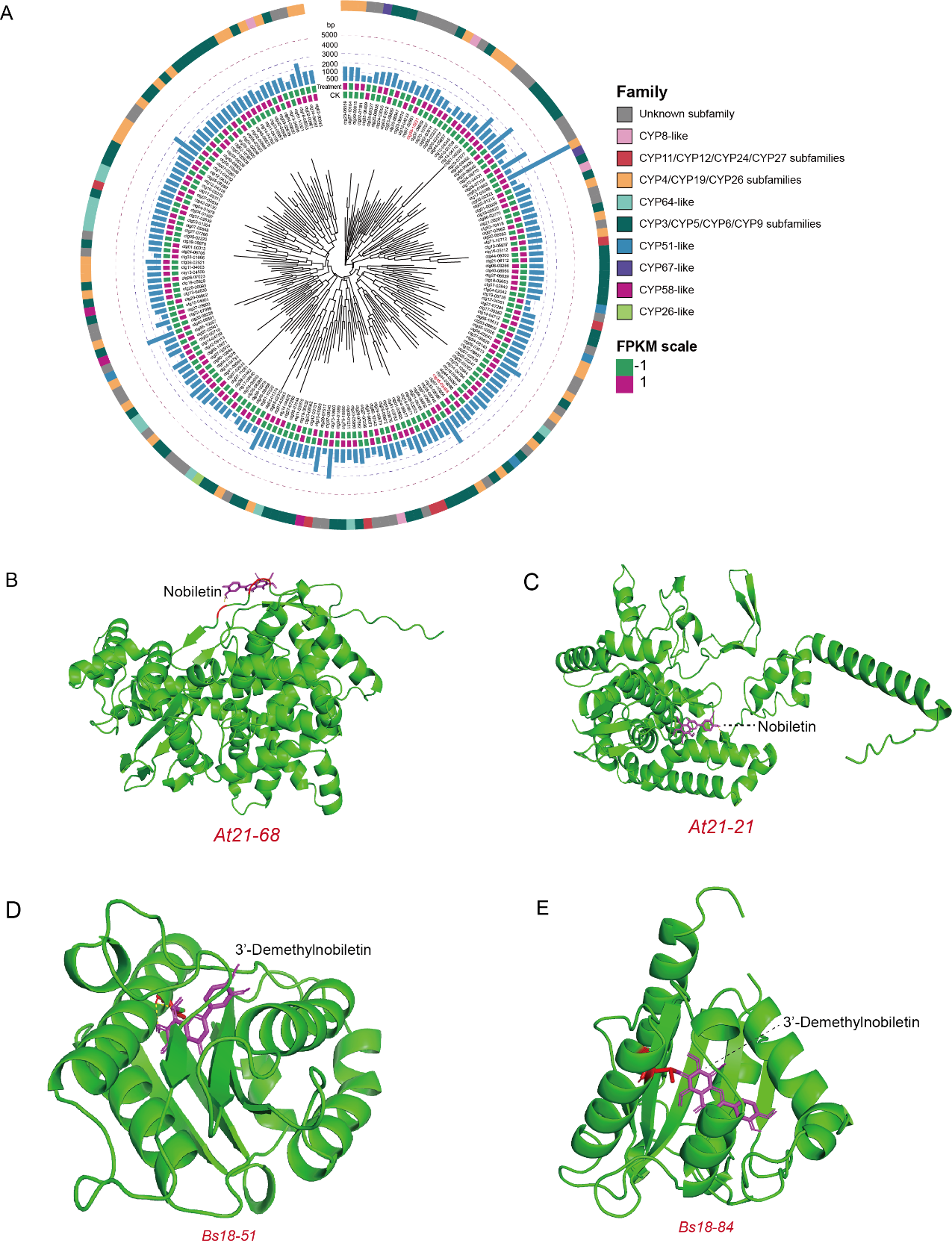


**Figure S16**


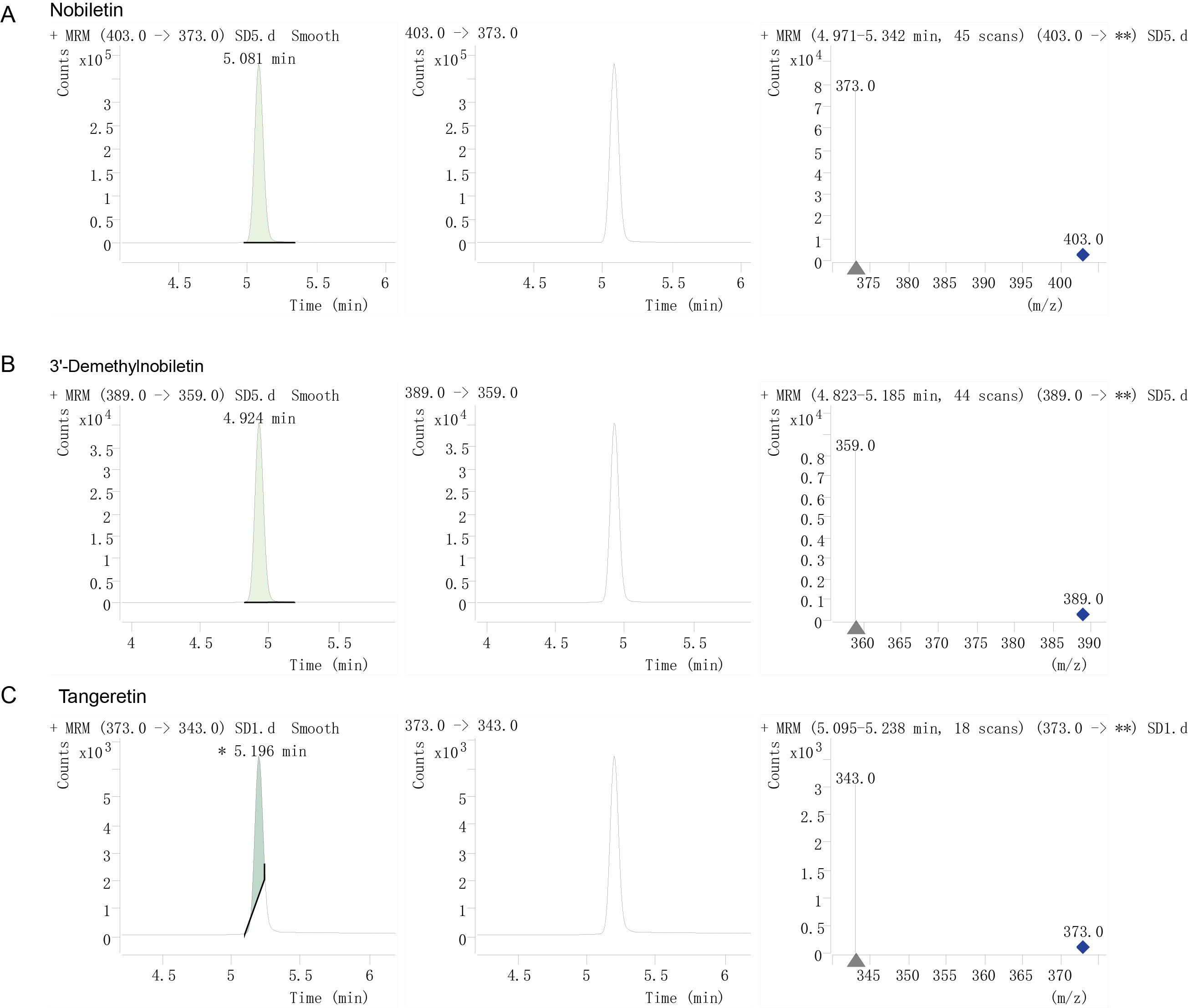


**Figure S17**


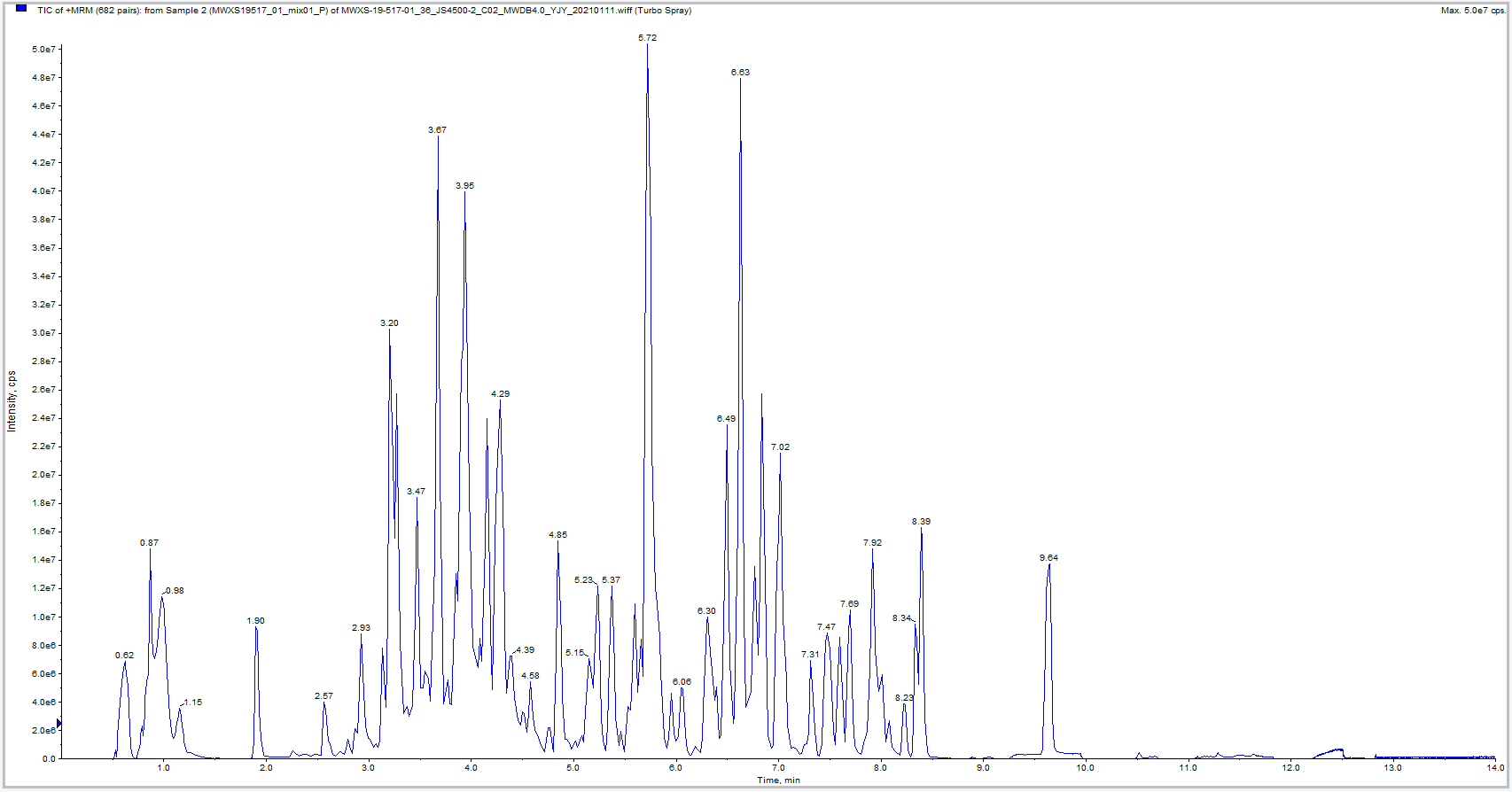


**Figure S18**


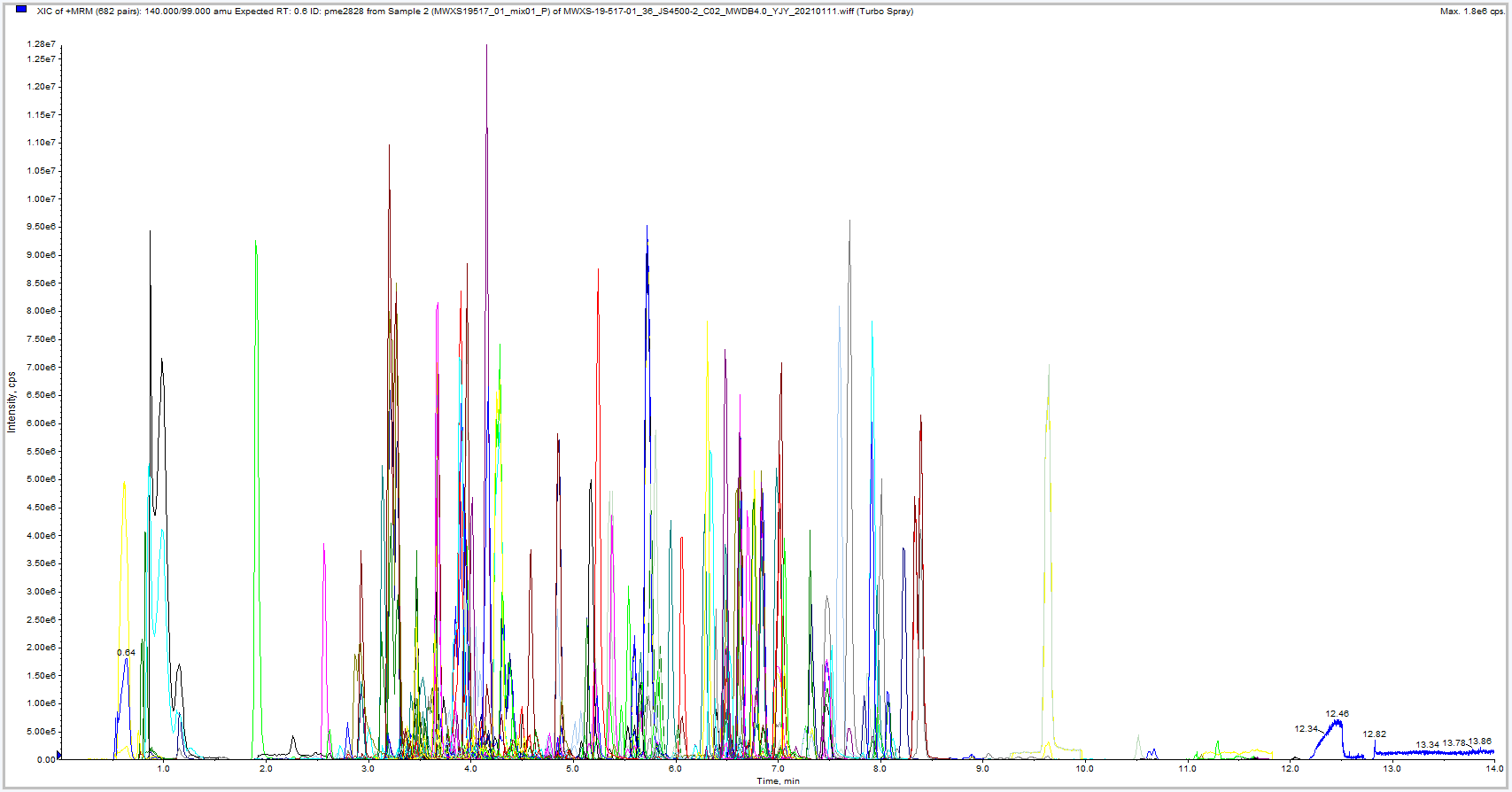


**Figure S19**


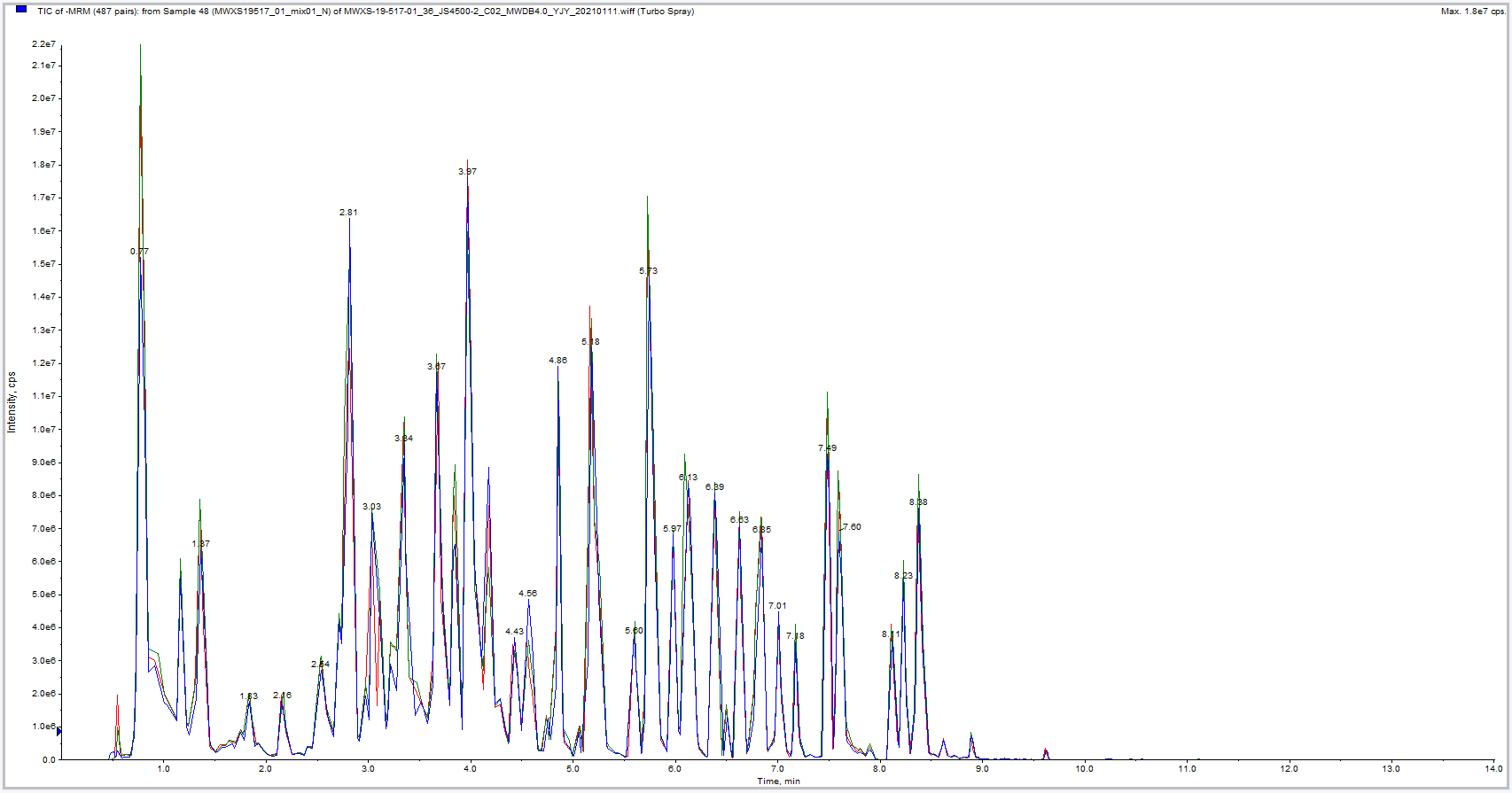

Supplement: Supplementary file 1 — Supporting File 1: advs74292‐sup‐0002‐SuppMat.docx. [file ADVS-13-e00267-s002.docx]
